# Development of a Humanized Antibody with High Therapeutic Potential against Dengue Virus Type 2

**DOI:** 10.1371/journal.pntd.0001636

**Published:** 2012-05-01

**Authors:** Pi-Chun Li, Mei-Ying Liao, Ping-Chang Cheng, Jian-Jong Liang, I-Ju Liu, Chien-Yu Chiu, Yi-Ling Lin, Gwong-Jen J. Chang, Han-Chung Wu

**Affiliations:** 1 Graduate Institute of Life Sciences, National Defense Medical Center, Taipei, Taiwan; 2 Institute of Cellular and Organismic Biology, Academia Sinica, Taipei, Taiwan; 3 Institute of Biomedical Sciences, Academia Sinica, Taipei, Taiwan; 4 Arbovirus Diseases Branch, Division of Vector-Borne Infectious Diseases, Centers for Disease Control and Prevention, Public Health Service, United States Department of Health and Human Services, Fort Collins, Colorado, United States of America; University of North Carolina at Chapel Hill, United States of America

## Abstract

**Background:**

Dengue virus (DENV) is a significant public health threat in tropical and subtropical regions of the world. A therapeutic antibody against the viral envelope (E) protein represents a promising immunotherapy for disease control.

**Methodology/Principal Findings:**

We generated seventeen novel mouse monoclonal antibodies (mAbs) with high reactivity against E protein of dengue virus type 2 (DENV-2). The mAbs were further dissected using recombinant E protein domain I-II (E-DI-II) and III (E-DIII) of DENV-2. Using plaque reduction neutralization test (PRNT) and mouse protection assay with lethal doses of DENV-2, we identified four serotype-specific mAbs that had high neutralizing activity against DENV-2 infection. Of the four, E-DIII targeting mAb DB32-6 was the strongest neutralizing mAb against diverse DENV-2 strains. Using phage display and virus-like particles (VLPs) we found that residue K310 in the E-DIII A-strand was key to mAb DB32-6 binding E-DIII. We successfully converted DB32-6 to a humanized version that retained potency for the neutralization of DENV-2 and did not enhance the viral infection. The DB32-6 showed therapeutic efficacy against mortality induced by different strains of DENV-2 in two mouse models even in post-exposure trials.

**Conclusions/Significance:**

We used novel epitope mapping strategies, by combining phage display with VLPs, to identify the important A-strand epitopes with strong neutralizing activity. This study introduced potential therapeutic antibodies that might be capable of providing broad protection against diverse DENV-2 infections without enhancing activity in humans.

## Author Summary

Dengue virus (DENV) infection remains a serious health threat despite the availability of supportive care in modern medicine. Monoclonal antibodies (mAbs) of DENV would be powerful research tools for antiviral development, diagnosis and pathological investigations. Here we described generation and characterization of seventeen mAbs with high reactivity for E protein of DENV. Four of these mAbs showed high neutralizing activity against DENV-2 infection in mice. The monoclonal antibody mAb DB32-6 showed the strongest neutralizing activity against diverse DENV-2 and protected DENV-2-infected mice against mortality in therapeutic models. We identified neutralizing epitopes of DENV located at residues K310 and E311 of viral envelope protein domain III (E-DIII) through the combination of biological and molecular strategies. Comparing the strong neutralizing activity of mAbs targeting A-strand with mAbs targeting lateral ridge, we found that epitopes located in A-strand induced stronger neutralizing activity than those located on the lateral ridge. DB32-6 humanized version was successfully developed. Humanized DB32-6 variant retained neutralizing activity and prevented DENV infection. Understanding the epitope-based antibody-mediated neutralization is crucial to controlling dengue infection. Additionally, this study also introduces a novel humanized mAb as a candidate for therapy of dengue patients.

## Introduction

Dengue is the most important arthropod-borne viral disease in humans and an increasing public health concern in tropical and subtropical regions of the world. Approximately 50–100 million cases of dengue fever (DF) and 500,000 cases of dengue hemorrhagic fever (DHF) occur every year, and 2.5 billion people are at risk of dengue infection globally [1], [2]. Dengue infection may lead to fever, headache and joint pain in milder cases but may also lead to the more severe life-threatening DHF/dengue shock syndrome (DSS) has plasma leakage, thrombocytopenia, and hemorrhagic manifestations, possibly leading to shock [3], [4].

Dengue virus (DENV) is positive-sense single-stranded RNA virus of approximately 11 kb genome of the genus *Flavivirus*, a family *Flaviviridae*. It has four genetically and antigenically related viral serotypes: DENV-1, -2, -3 and -4. Flaviviruses encode a single polyprotein processed by host and viral protease to produce three structural proteins, including capsid (C) protein, precursor membrane/membrane (prM/M) and envelope (E) protein, and seven nonstructural proteins: NS1, NS2A, NS2B, NS3, NS4A, NS4B and NS5 [5]. The E protein, a 53 kDa glycoprotein important for attachment, entry, and viral envelope fusion, can bind to cellular receptors and induce neutralizing antibodies [6], [7].

The DENV consists of an icosahedral ectodomain, containing 180 copies of the E protein [8]. E protein monomer contains three structural and functional domains [9], [10]. E protein domain I (E-DI) is a central β-barrel structure. E protein domain II (E-DII) is organized into two long finger-like structures and contains the flaviviruses conserved fusion loop. E protein domain III (E-DIII) has an immunoglobulin-like fold and may mediate interactions between the virus and the receptors on the host cell [11]. Studies of the biological characteristics and epitope specificities of mouse monoclonal antibodies (mAbs) have elucidated the antigenic structure of flavivirus E proteins [12]–[15]. Serotype-specific mAbs with neutralizing activity against DENV-2 have been found to be located on the lateral ridge of E-DIII and the subcomplex-specific mAbs recognized A-strand of E-DIII [14], [16], [17]. Antibody-mediated neutralization has been found to alter the arrangement of viral surface glycoproteins that prevent cells from viral attachment [16]. Binding of an antibody to the viral surface can interfere with virus internalization or membrane fusion [6].

Primary DENV infection is believed to provide lifelong immunity against re-infection with the same serotype [18], [19]. However, humoral immune responses to DENV infection are complex [20]–[22], and may exacerbate the disease during heterologous virus infection [18], [19]. Antibody-dependent enhancement (ADE) in dengue pathogenesis results from the increase in the efficiency of virus infection in the presence of non-neutralizing or sub-neutralizing concentrations of anti-E or anti-prM immunoglobulins [21], [23]. The attachment of antibody-virus complex to such Fcγ receptor-bearing cells as monocytes and macrophages can lead to an increased virus replication [18], [24], [25].

A better understanding of the neutralizing epitopes may facilitate the generation of new antibody-based therapeutics against DENV infection. In this study, we generated several mAbs against DENV-2. We found that serotype-specific anti-E-DIII mAbs played an important role in the neutralization of virus infectivity. Studies of the neutralizing epitopes found the strongest mAbs to be DB32-6 and DB25-2, both DENV-2 serotype-specific antibodies. These two mAbs recognized the A-strand of E-DIII at residues K310 and E311, respectively. Humanized DB32-6 mAb efficiently neutralized DENV-2 infection in a therapeutic mouse model and its variant version prevented enhancing activity.

## Methods

### Cells and viruses

BHK-21 cells were grown at 37°C with 5% CO_2_ in Minimal Essential Medium (MEM, Gibco) supplemented with 10% heat-inactivated fetal bovine serum (FBS, Gibco) and 100 U/ml penicillin, 100 µg/ml streptomycin, 0.25 µg/ml amphotericin B (Antibiotic-Antimycotic, Gibco). *Aedes albopictus* C6/36 cells were grown at 28°C in 1∶1 Mitsuhashi and Maramorosch (MM) insect medium (Sigma-Aldrich)/Dulbecco's modified Eagle's medium (DMEM, Gibco) containing 10% FBS and 100 U/ml penicillin, 100 µg/ml streptomycin, 0.25 µg/ml amphotericin B (Antibiotic-Antimycotic, Gibco). The four DENVs (DENV-1 Hawaii, DENV-2 16681, DENV-3 H87 and DENV-4 H241) were provided by Dr. Duane J. Gubler from the Centers for Disease Control and Prevention, Fort Collins, U.S.A. The various DENV-2 strains including New Guinea-C (NGC), NGC-N (mouse-adapted neurovirulent), PL046 and Malaysia 07587 were used in this study [26], [27]. These viruses were passaged in C6/36 cells.

### Generation and purification of mAbs

Anti-DENV-2 mAbs were generated according to previously described procedures [28], [29]. Female 4- to 6-week-old BALB/c mice were immunized with 10^7^ plaque-forming units (pfu) of DENV-2 (16681). The DENV-2 was purified from viral culture supernatant using 4G2 (an anti-E protein mAb)-coupled protein G-Sepharose 4 Fast Flow gel. After four inoculations with the same concentration of antigens, the splenocytes from the immunized mouse spleen were harvested and then fused with mouse myeloma NS-1 cells. Fused cells were cultured in DMEM supplemented with 15% FBS, HAT medium and hybridoma cloning factor (Roche) in 96-well tissue culture plates. Two weeks after fusion, culture supernatants were screened by ELISA. Selected clones were subcloned by limiting dilutions. Hybridoma clones were isotyped using a commercially isotyping kit (Southern Biotech) by ELISA. Ascites fluids were produced in pristine-primed BALB/c mice. mAbs were affinity-purified by standard protein G-Sepharose 4 Fast Flow (GE Healthcare Bio-Sciences) according to manufacturer's directions.

### Screening of mAbs against DENV1-4 by ELISA

C6/36 cells at 80% confluency in 96-well plates were infected with DENV-1 to -4 to produce viral antigens. These cells were then harvested 5–7 days after infection. One µg/ml mAbs was added to the plates and incubated at room temperature (RT) for 1 h. After washing with PBS, horseradish peroxidase (HRP)-conjugated anti-mouse IgG (Jackson ImmunoResearch Laboratories) was incubated at RT for 1 h. Finally, plates were incubated with peroxidase substrate *o*-phenylenediamine dihydrochloride (OPD; Sigma-Aldrich). Reaction was stopped with 3N HCl and optical density was measured using a microplate reader set at 490 nm.

### Western blot analysis

C6/36 cells were harvested after viral infection. Lysates or expression proteins were collected. Cell extracts were mixed with sample buffer (Bio-Rad Laboratories). Protein samples were separated by SDS-PAGE and transferred to nitrocellulose membrane (Hybond-C Super). Nonspecific antibody-binding sites were blocked with 5% skimmed milk in PBS, and membranes were incubated with primary antibody. Blot was then treated with horseradish peroxidase-conjugated goat anti-mouse immunoglobulin (Jackson ImmunoResearch Laboratories) and then developed with enhanced chemiluminescence reagents (ECL, Thermo Fisher Scientific).

### Immunofluorescence assay (IFA)

BHK-21 cells at 80% confluency were infected at a multiplicity of infection (MOI) of 0.5 with DENV-2 (16681). After 2 days infection, the cells were fixed with 1∶1 methanol/acetone for 10 min at −20°C. Cells were blocked using PBS supplemented with 1% BSA for 1 h at RT. Primary anti-DENV antibodies or control antibodies (normal mouse IgG, Jackson ImmunoResearch Laboratories) were diluted (1∶250) in block solution for 1 h at RT. Secondary antibody, FITC-conjugated goat anti-mouse IgG (Jackson ImmunoResearch Laboratories) was diluted to 1∶250 and supplemented with DAPI (Invitrogen) diluted 1∶2,000 for 1 h at RT. The binding activity of antibodies to the DENV-2-infected or transfected cells were observed and photographed through a fluorescence microscope.

### Cloning and expression of DENV recombinant proteins

The expression constructs of E-DI-II and E-DIII were cloned into the pET21a vector (Merck). The E-DI-II, comprising amino acids 1–295 of the E protein, was tagged to flag and hexahistidine at the C terminus for affinity purification. The E-DIII, comprising amino acids 295–400 of the E protein, was tagged to flag and hexahistidine, too. The plasmids were expressed in *Escherichia coli* strain BL21 (DE3). The recombinant proteins E-DI-II and E-DIII were analyzed using 12% SDS-PAGE by Western blot analysis. The DNA fragments corresponding to E-DI-II and E-DIII were also cloned into a mammalian expression vector, pcDNA3.1 (Invitrogen). The expression constructs of DENV-2 C, prM, prM-E, E, NS1, NS2A, NS2B, NS2B-3, NS3, NS4A, NS4B and NS5 were obtained from Dr. Y.-L. Lin [30]. Transient expression of DENV-2 proteins in BHK-21 cells was transfected by PolyJet (SignaGen Laboratories) according to manufacturer's recommendations and then to test specificity of mAbs.

### *In vitro* neutralization assay

(i) For the plaque reduction neutralization test (PRNT), eight 3-fold serial dilutions of mAbs (from 200 µg/ml to 0.1 µg/ml) were mixed with an equal volume of 200 pfu of DENV-2 (16681) and incubated at 4°C for 1 h. The final concentration of mAbs at the PRNT ranged from 100 to 0.05 µg/ml. Antibody-virus mixtures (100 µl) were added to BHK-21 cells at 80%–90% confluency in 12-well plates. After absorption of virus for 2 h, BHK-21 cells were washed and 2 ml of 1% (w/v) carboxyl methyl cellulose (Sigma-Aldrich) in MEM plus 2% (v/v) FBS was layered onto the infected cells. After incubation at 37°C for 5 to 7 days, the viral plaque that had formed on the cell monolayer was fixed by 1 ml 3.7% formaldehyde (Sigma-Aldrich) at RT for 1 h. The cells were then stained with 1% crystal violet. Percentage of plaque reduction was calculated as: %Inhibition = 100−[(plaque number incubated with mAb/plaque number without mAb)×100]. (ii) For flow cytometry, serial dilutions of DB32-6 mAb were incubated with DENV-2 (16681, NGC, PL046 and Malaysia 07587) at MOI of 0.5 at 4°C 1 h before adding BHK-21 cells. After 2 h absorption, the monolayers were washed and incubated with MEM (Gibco) plus 2% (v/v) FBS at 37°C for 2 days. The cells infected with DENV-2 were washed and fixed with 3.7% formaldehyde at 4°C for 10 min. They were then permeabilized in PBS supplemented with 1% FBS, 0.1% saponin (Sigma) at 4°C for 10 min. For staining, cells were incubated with 4G2 at a concentration of 1 µg/ml at 4°C for 30 min. After two washes, R-Phycoerythrin (PE)-conjugated AffiniPure F(ab′)_2_ fragment goat anti-mouse IgG (H+L) (Jackson ImmunoResearch Laboratorie) diluted 1∶250 was then added at 4°C for 30 min followed by two washes and analyzed by flow cytometry. % Infection = (the intensity of cells incubated with mAb/without mAb)×100.

### Mouse experiments

This study was carried out following strict guidelines from the care and use manual of National Laboratory Animal Center. The protocol was approved by the Committee on the Ethics of Animal Experiments of Academia Sinica. (Permit Number: MMiZOOWH2009102). The mice were killed with 50% CO_2_ containing 50% O_2_. All efforts were made to minimize suffering. (i) Breeder mice of the ICR strain were purchased from the Laboratory Animal Center National Taiwan University College of Medicine. Purified mAbs at doses of 1, 10 and 100 µg/ml were incubated with 1×10^4^ pfu (25-fold LD_50_) of DENV-2 (16681) at 4°C for 30 mins. Two-day-old suckling mouse brain was inoculated with 20 µl of the reaction mixture by intracranial (i.c.) injection. Survival rate and signs of illness, including paralysis, were observed daily for 21 days following challenge. In post-exposure therapeutic experiments, mice were passively injected with 5 µg of mAb via i.c. route after 1 day of infection. (ii) *Stat1*-deficient mice *(Stat1^−/−^)* [31] were bred in the specific-pathogen-free animal facility at the Institute of Biomedical Sciences, Academia Sinica. Mice were challenged intraperitoneally with 1×10^5^ pfu (300-fold LD_50_) of DENV-2 (NGC-N) in 300 µl of PBS and simultaneously injected intracranially (i.c.) with 30 µl of PBS. In prophylaxis experiments, antibodies (100 µg per mouse, intraperitoneally) were administered 1 day before infection and administered on day 0, 1, 3, 5 and 7 after infection. In postexposure therapeutic experiments, antibodies (100 µg per mouse, intraperitoneally) were administered on day 1, 3, 5 and 7 after infection.

### Phage display biopanning

The phage display biopanning procedures were performed according to previous reports [28], [32]. Briefly, an ELISA plate was coated with mAbs at 100 µg/ml. Samples of 100 µl diluted mAb were then added to wells and incubated at 4°C for 6 h. After washing and blocking, the phage-displayed peptide library (New England BioLabs, Inc.) was diluted to 4×10^10^ pfu of phage and incubated for 50 mins at RT. After washing, bound phage was eluted with 100 µl 0.2 M glycine/HCl (pH 2.2) and neutralized with 15 µl 1 M Tris/HCl (pH 9.1). The eluted phage was amplified in ER2738 for subsequent rounds of selection. The phage was titrated onto LB medium plates containing IPTG and X-Gal. The biopanning protocol for the second and third rounds was identical to the first round except for the addition of 2×10^11^ pfu of amplified phage for biopanning.

### Identification of immunopositive phage clones by ELISA

An ELISA plate was coated with 50 µl mAbs 50 µg/ml. After washing and blocking, amplified phage diluted 5-fold was added to coated plate and incubated at RT for 1 h. After washing, 1∶5000 diluted HRP-conjugated anti-M13 antibody (GE Healthcare) was added at RT for 1 h. OPD developed and was terminated with HCl. Optical density was measured at 490 nm.

### Identification of neutralizing epitopes by virus-like particle (VLP) mutants

We used the recombinant expression plasmid pCBD2-2J-2-9-1 [33] to generate VLP mutants. Various VLP mutants were generated by site-directed mutagenesis derived from pCBD2-2J-2-9-1 as a template. PCR was performed using pfu ultra DNA polymerase (MERCK) and all mutant constructs were confirmed by sequencing. BHK-21 cells at 80%-90% confluency in 48-well plates were transfected with plasmids of various VLPs. After two days transfection, the cells were washed with PBS supplemented with 1% FBS, fixed with 3.7% formaldehyde at 4°C for 10 min, and then permeabilized in PBS supplemented with 1% FBS, 0.1% saponin (Sigma-Aldrich) at 4°C for 10 min. For staining, cells were incubated with mAbs at 4°C for 30 min, DB32-6, DB25-2, 3H5 and mix mAbs (4G2, DB2-3, DB13-19, DB21-6 and DB42-3) at a concentration of 0.1, 1, 1 and 1 µg/ml, respectively. After being washed twice, R-Phycoerythrin (PE)-conjugated AffiniPure F(ab′)_2_ fragment goat anti-mouse IgG (H+L) (Jackson ImmunoResearch Laboratories) diluted to 1∶250 was then added at 4°C for 30 min and analyzed by flow cytometry. Relative recognition was performed according to previously described procedures and calculated as [intensity of mutant VLP/intensity of WT VLP] (recognized by a mAb)×[intensity of WT VLP/intensity of mutant VLP] (recognized by mixed mAbs) [34].

### Cloning and sequencing of neutralizing mAbs

Total RNA was extracted from hybridoma cells using the TRIzol reagent (Invitrogen) and mRNA was isolated with the NucleoTrap mRNA Mini Kit (Macherey-Nagel GmbH & Co. KG.). Purified mRNA was reverse transcribed using oligo (dT) as a primer in a ThermoScript RT-PCR system (Invitrogen). The variable heavy- and light-chain domains (V_H_ and V_L_) were amplified from the cDNA product by PCR with a variety of primer sets [35], [36]. The PCR products were cloned using the TA kit (Promega) and the V_H_ and V_L_ sequences were determined by DNA sequencing. Software Vector NTI was used for sequence analysis. From these sequences, the framework regions (FRs) and complementarity-determining regions (CDRs) were analyzed by comparing them with those found in the Kabat database and the ImMunoGeneTics database [37].

### Construction and expression of humanized DB32-6

Two human genes, GenBank accession DI084180 and DI075739, were 94.7% and 92.2% identical to DB32-6 V_H_ and V_L_, respectively. Humanized DB32-6 V_H_ consisted of the modified FR1 to FR4 from the accession DI084180 gene, and the CDR1 to CDR3 of the DB32-6 V_H_, respectively, while humanized DB32-6 V_L_ consisted of the modified FRs from the accession DI075739 gene and the CDRs of the DB32-6 V_L_. Both were synthesized (GENEART) and amplified by PCR using pfu Turbo DNA polymerase (EMD Bioscience). The resulting V_H_ was cloned into modified expression vector pcDNA3.1 (Invitrogen) with a signal peptide and human IgG1 constant region, while the V_L_ was cloned into modified expression vector pSecTag (Invitrogen). We generated a variant of humanized DB32-6 (hDB32-6 variant) in which leucine residues at positions 1.2 and 1.3 of C_H_2 domain were substituted with alanine residues [38]. The V_H_ and V_L_ plasmids were cotransfected into CHO-K1 cells and selected by G 418 and puromycin for 2–3 weeks. Transformed cells were limit diluted in 96-well plates. After two weeks, stable clones produced humanized antibodies in the McCoy's 5A medium (Sigma-Aldrich), as identified by ELISA. Humanized antibodies were produced by CELLine AD 1000 (INTEGRA Biosciences) according to manufacturer's directions.

### Surface plasmon resonance

Murine and humanized DB32-6 mAbs affinity analysis for E-DIII of DENV-2 was performed by surface plasmon resonance (BIAcore X, Biacore, Inc). Purified E-DIII (50 µg/ml) was immobilized on a CM5 sensor chip (Biacore, Inc) and injected at a flow rate of 10 µl/min. The mAbs were diluted to 4, 2, 1, 0.5, 0.25 and 0 nM in HBS-EP buffer (Biacore, Inc). mAbs were injected at a flow rate of 30 µl/min for 3 min and then allowed to dissociate over 1.5 min. Regeneration of the surface was achieved with an injection of 10 mM glycine HCl/0.2 M NaCl (pH 3.0) before each mAb injection. The data were analyzed by the BIAevaluation software with a global fit 1∶1 binding model.

### Antibody-dependent enhancement (ADE) assay

Serial dilutions of mAbs were mixed with DENV-2 (16681) at MOI of 1 at 4°C for 1 h. The 100 µl mixture were incubated with 5×10^4^ K562 cells [39] in 96-well plates at 37°C for 2 h. After infection, the cells were washed and incubated with RPMI (Gibco) plus 2% (v/v) FBS at 37°C for 2 days. The cells were washed with PBS supplemented with 1% FBS, fixed with 3.7% formaldehyde, and permeabilized in PBS supplemented with 1% FBS, 0.1% saponin (Sigma) at 4°C for 10 min. For staining, cells were incubated with DB42-3 at a concentration of 3 µg/ml at 4°C for 30 min. After two times washes, R-Phycoerythrin (PE)-conjugated AffiniPure F(ab′)_2_ fragment goat anti-mouse IgG (H+L) (Jackson ImmunoResearch Laboratories, West Grove, PA) diluted 1∶250 was then added at 4°C for 30 min follow by two times wash steps and analyzed by flow cytometry.

### Statistical analysis

Survival rate was expressed using Kaplan-Meier survival curve, and log rank test was used to determine the significant differences. For body weight change experiments, paired t-test was used to determine the significant differences, * *P*<0.05, ** *P*<0.01.

## Results

### Generation and characterization of neutralizing mAbs against E protein of DENV-2

**Figure 1:**
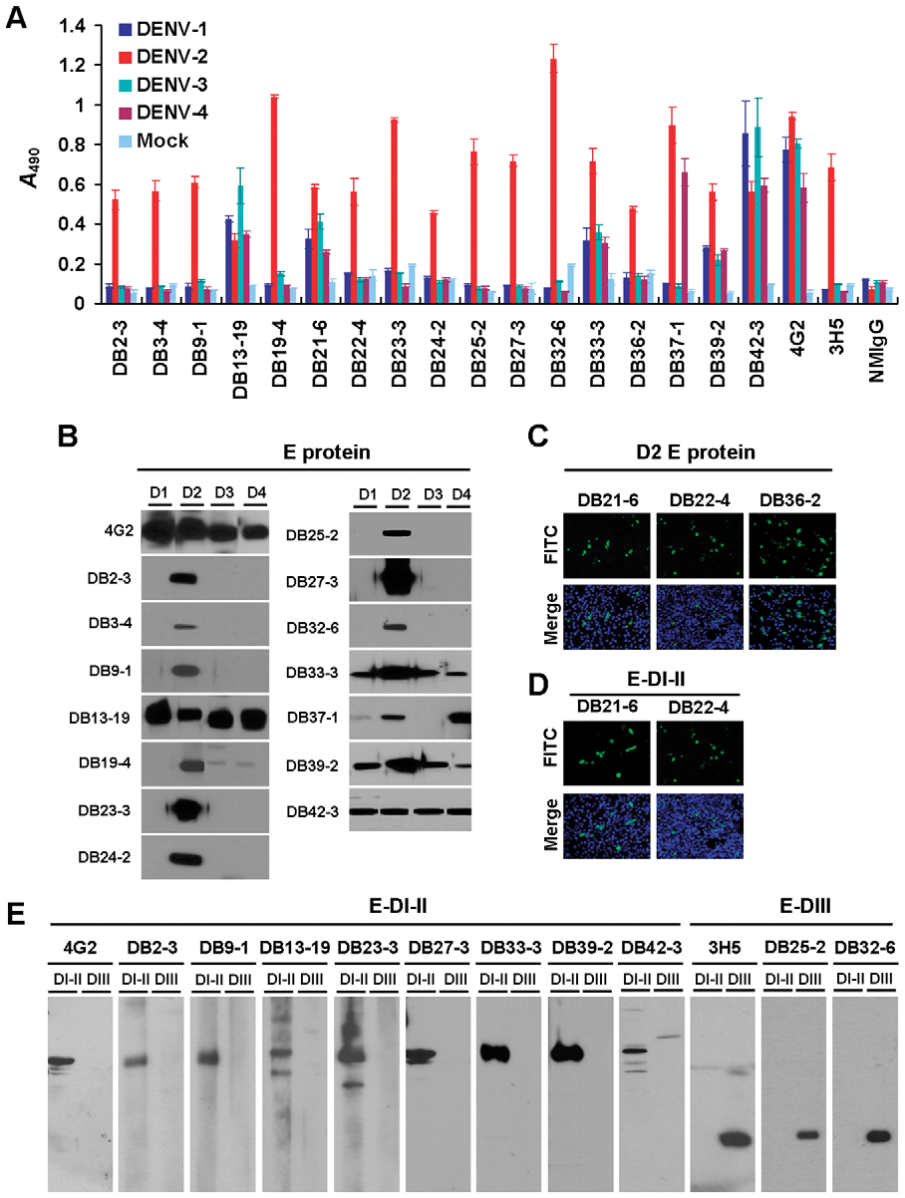
Characterization of mAbs against DENV. (A) C6/36 insect cells were infected by DENV-1, -2, -3 and -4 or uninfected (Mock). After fixation and permeabilization, mAbs were incubated with cells and binding was assessed by cellular ELISA. *A*_490_, optical density at 490 nm. (B) Identification of mAbs by Western blotting. C6/36 cells were infected with DENV-1 to -4 (D1, D2, D3 and D4) as viral antigens. Protein samples were dissolved in native sample buffer and fractionated by 10% SDS-PAGE. mAbs recognized E protein (53 kDa) of DENV. (C and D) mAbs recognized DENV-2 E protein and E-DI-II was determined by IFA, respectively. (E) Dissection of DENV-2 mAbs recognized E-DI-II or E-DIII by Western blot analysis. The DENV-2 recombinant E-DI-II-flag (36 kDa) and E-DIII-flag (17 kDa) fusion proteins were expressed in *Escherichia coli*. Protein extract was dissolved in denatured sample buffer and fractionated on 12% SDS-PAGE. 4G2, a cross-reactive mAb and 3H5, a DENV-2 serotype-specific mAb recognized D2-E-DI-II and D2-E-DIII, respectively. They were used as positive controls.

Seventeen mAbs with high reactivity against E protein of DENV-2 were generated after immunization of mice with DENV-2 strain 16681. We identified 17 mAbs belonging to the IgG isotype that reacted with DENV-2-infected cells but not with mock-infected cells using immunofluorescence assay (IFA) (Figure S1) and ELISA (Figure 1A). 4G2 was a pan-flavivirus mAb that could recognize the fusion loop of E-DI-II, and 3H5 (ATCC HB46) was a DENV-2 serotype-specific mAb that could recognize the lateral ridge of E-DIII [12], [17], [40]. Both 4G2 and 3H5 were used as positive controls (Figure 1). The specificities of the mAbs recognized as the four DENVs were further confirmed by ELISA and Western blotting (Figures 1A–1B and Table 1). Based on our Western blot analysis using a nonreducing condition, 14 of the mAbs recognized E protein (53 kDa) (Figure 1B). Three mAbs could not be identified by Western blotting. In order to identify the target proteins of these mAbs, we prepared BHK-21 cells transfected with plasmids expressing DENV-2 C, prM, prM-E, E, NS1, NS2A, NS2B, NS2B-3, NS3, NS4A, NS4B and NS5 (Figure S2). Results indicated that three mAbs (DB21-6, DB22-4 and DB36-2) recognized E protein (Figure 1C). The identification and characterization of the 17 mAbs are summarized in Table 1.

**Table 1:** Characterization of DENV-2 mAbs by IFA, ELISA, WB and PRNT_50_ (µg/ml).

mAbs	Isotype, Light chain	Specificity	IFA	ELISA				WB				PRNT_50_ (µg/ml)
			D2	D1	D2	D3	D4	D1	D2	D3	D4	D2
DB2-3	IgG1, κ	E-DI-II	+	−	+	−	−	−	+	−	−	≦1.2
DB3-4	IgG1, κ	E	+	−	+	−	−	−	+	−	−	≦3.7
DB9-1	IgG1, κ	E-DI-II	+	−	+	−	−	−	+	−	−	≦3.7
DB13-19	IgG1, κ	E-DI-II	+	+	+	+	+	+	+	+	+	≦33
DB19-4	IgG2b, κ	E	+	−	+	−	−	−	+	−	−	≦3.7
DB21-6	IgG1, κ	E-DI-II	+	+	+	+	+	−	−	−	−	>33
DB22-4	IgG2a, κ	E-DI-II	+	−	+	−	−	−	−	−	−	>33
DB23-3	IgG2a, κ	E-DI-II	+	−	+	−	−	−	+	−	−	≦0.41
DB24-2	IgG2a, κ	E	+	−	+	−	−	−	+	−	−	≦3.7
DB25-2	IgG1, κ	E-DIII	+	−	+	−	−	−	+	−	−	≦1.2
DB27-3	IgG1, κ	E-DI-II	+	−	+	−	−	−	+	−	−	>33
DB32-6	IgG2b, κ	E-DIII	+	−	+	−	−	−	+	−	−	≦0.14
DB33-3	IgG1, κ	E-DI-II	+	+	+	+	+	+	+	+	+	>33
DB36-2	IgG1, κ	E	+	−	+	−	−	−	−	−	−	n.d.
DB37-1	IgG1, κ	E	+	−	+	−	+	−	+	−	+	>33
DB39-2	IgG1, κ	E-DI-II	+	+	+	+	+	+	+	+	+	>33
DB42-3	IgG1, λ	E-DI-II	+	+	+	+	+	+	+	+	+	≦3.7
3H5	IgG1	E-DIII	+	−	+	−	−	−	+	−	−	≦0.41
4G2	IgG2a	E-DI-II	+	+	+	+	+	+	+	+	+	≦11

mAbs, monoclonal antibodies; IFA, immunofluorescence assay; ELISA, enzyme-linked immunosorbent assay; WB, Western blotting; PRNT, plaque reduction neutralization test; D1, D2, D3, and D4, DENV-1 to -4; Ig, immunoglobulin; E, envelope protein; E-DI-II, envelope protein domain I-II; E-DIII, envelope protein domain III. (+) positive result to DENV, *A*_490_>0.2; (−) negative result to DENV, *A*_490_<0.2; (n.d.) not determined.

To characterize the antigenic structure of the DENV E protein and to study the relationship between epitopes and their neutralizing potency, we constructed and expressed the recombinant E-DI-II and E-DIII from DENV-2 in *E. coli* and mammalian expression systems. Western blot analysis and IFA showed that, of the 17 mAbs recognizing E protein, 10 mAbs (DB2-3, DB9-1, DB13-19, DB21-6, DB22-4, DB23-3, DB27-3, DB33-3, DB39-2 and DB42-3) targeted to E-DI-II and 2 mAbs (DB25-2 and DB32-6) recognized E-DIII (Figures 1D–1E and Table 1). However, 5 mAbs could not be identified by these two assays.

**Figure 2:**
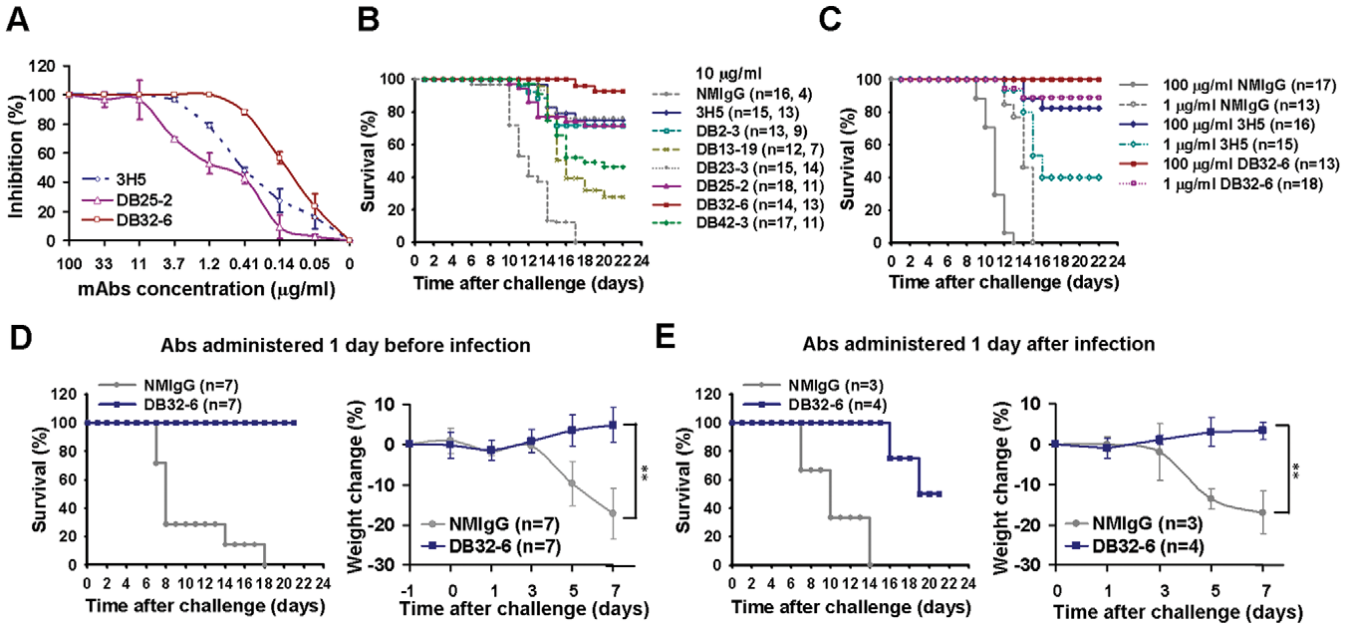
Neutralizing activity of DENV-2 mAbs and DB32-6 show therapeutic efficacy in *Stat1^−/−^* mice. (A) Plaque reduction neutralization test with purified mAbs (3H5, DB25-2 and DB32-6) against the DENV-2 (16681). The data for three independent experiments are shown. (B and C) Neutralizing mAbs protected against DENV-2-induced lethality in suckling mice. (B) Ten µg/ml of neutralizing mAbs (DB2-3, DB13-19, DB23-3, DB25-2, DB32-6, DB42-3 and 3H5) and NMIgG were incubated with 1×10^4^ pfu (25-fold LD_50_) of DENV-2 (16681) at 4°C for 30 min before injection. Two-day-old suckling mice (ICR strain) were challenged intracranially (i.c.) with the mAb:DENV-2 mixture. Mice were observed daily for signs of illness including paralysis for 21 days. The data for each group were an average of two independent experiments. (C) DB32-6, 3H5 and NMIgG at 1 or 100 µg/ml were incubated with 1×10^4^ pfu (25-fold LD_50_) of DENV-2 (16681) and challenged via an i.c. route. Mice were observed daily for 21 days. (D) *Stat1*-deficient mice were challenged intraperitoneally (i.p.) with 1×10^5^ pfu (300-fold LD_50_) of DENV-2 (NGC-N). Antibodies (100 µg per mouse, i.p.) were administered 1 day before infection and administered again on day 0, 1, 3, 5 and 7 after infection (D left). Body weight change of mice treated with NMIgG or DB32-6 at various time-points after infection (D right). In the therapeutic experiments, antibodies (100 µg per mouse, i.p.) were administered on day 1, 3, 5 and 7 after infection (E left). Body weight change of mice (E right). Paired t-test was used to determine significance in body weight change, ** *P*<0.01. (B–E) Log rank test was used to determine significant differences in survival rate, and the mAbs with neutralizing activity were compared to the control group treated with NMIgG, *P*<0.001.

We evaluated the ability of mAbs to inhibit DENV-2 infection in BHK-21 cells using a plaque reduction neutralization test (PRNT). Ten mAbs had neutralizing activity with 50% PRNT (PRNT_50_) concentrations ranging from 0.14 µg/ml to 33 µg/ml (Table 1). DB32-6 was found to be a DENV-2 serotype-specific mAb against E-DIII (Figures 1A, 1B and 1E) and was the most efficient at neutralizing DENV-2 infection at a PRNT_50_ concentration of 0.14 µg/ml (Figure 2A). In addition, it could completely inhibit the infection at a lower concentration of 1.2 µg/ml (Figure 2A). The mAb DB25-2 was found to be a DENV-2 serotype-specific mAb against E-DIII (Figures 1A, 1B and 1E) and to neutralize DENV-2 at a PRNT_50_ titer of 1.2 µg/ml (Figure 2A). These findings indicate that serotype-specific mAb DB32-6 against E-DIII was the most potent in neutralizing DENV infection. Some serotype-specific mAbs, such as DB2-3 and DB23-3 against E-DI-II and DB25-2 against E-DIII showed strong neutralizing activity. Many complex reactive mAbs showed moderate-to-poor neutralizing activity (Table 1).

### mAbs prevent DENV-2-induced lethality in suckling mice and *Stat1^−/−^* mice

Two different mouse models were used to assess whether DB32-6 could efficiently protect mice against DENV-2 challenge. Protection assay of neutralizing mAbs was performed with ICR strain 2-day-old suckling mice [41]. Mice were inoculated intracerebrally with 20 µl of DENV-2-mAb mixture containing 1×10^4^ pfu (25-fold LD_50_) of DENV-2 with neutralizing mAbs at concentrations of 1, 10 or 100 µg/ml. Generally, the non-neutralizing antibody normal mouse IgG (NMIgG) treated group showed paralysis, ruffling, and slowing of activity around 6 to 9 days. This was followed by severe sickness leading to anorexia, asthenia and death within 9 to 17 days (Figures 2B and 2C). In contrast, mAbs DB32-6 at a concentration of 10 µg/ml protected 93% of the mice from the lethal challenge of DENV-2 (Figure 2B). mAbs 3H5, DB23-3, DB2-3 and DB25-2 had survival rates of 75%, 76%, 72% and 71%, respectively. DB42-3 and DB13-19 had survival rates of 46% and 28%, respectively (Figure 2B). The neutralizing mAbs showed a significant delay of the onset of paralysis and death relative to the NMIgG. To evaluate the therapeutic potential of the highly protective mAb DB32-6, we administered 100 µg/ml or 1 µg/ml to infected suckling mice. The survival rates for DB32-6 at 100 µg/ml or 1 µg/ml were 100% and 89%, respectively (Figure 2C). In comparison, 3H5 showed 82% and 40% survival rates at 100 µg/ml or 1 µg/ml, respectively.

*Stat1^−/−^* mice, which lack a transcription factor involved in interferons (IFNs) signaling were sensitive to lethality induced by DENV-2 infection [27], [31]. To test the potential therapeutic effects of the strongest neutralizing mAb DB32-6, we challenged *Stat1^−/−^* mice at a strict condition with 1×10^5^ pfu (300-fold LD_50_) of DENV-2 (NGC-N). After 21 days observation, mice showed ruffled fur, mild paralysis and lost approximate 20% of their initial body weight at day 7 after infection (*P*<0.01), and then they all died within 7–18 days of infection (Figures 2D and 2E). In the prophylaxis experiments, antibodies (100 µg per mouse, intraperitoneally) were administered 1 day before infection and at day 0, 1, 3, 5 and 7 after infection. The DB32-6 prophylactically treated group showed 100% protection (Figure 2D left). Even in the postexposure therapeutic experiments, the DB32-6 treated mice had a survival rate of 50% (Figure 2E left). The mAb DB32-6 had excellent neutralizing activity against different DENV-2 strains (16681 and NGC-N) in two mouse models.

To further evaluate whether the strongest mAb DB32-6 could broadly neutralize the diverse DENV-2 strains, we infected BHK-21 cells with four different DENV-2 Southeast Asian genotype strains, 16681, NGC, PL046 and Malaysia 07587. Remarkably, mAb DB32-6 exhibited effective neutralization against various DENV-2 strains (Figure S3).

### Identification of neutralizing epitopes

**Figure 3:**
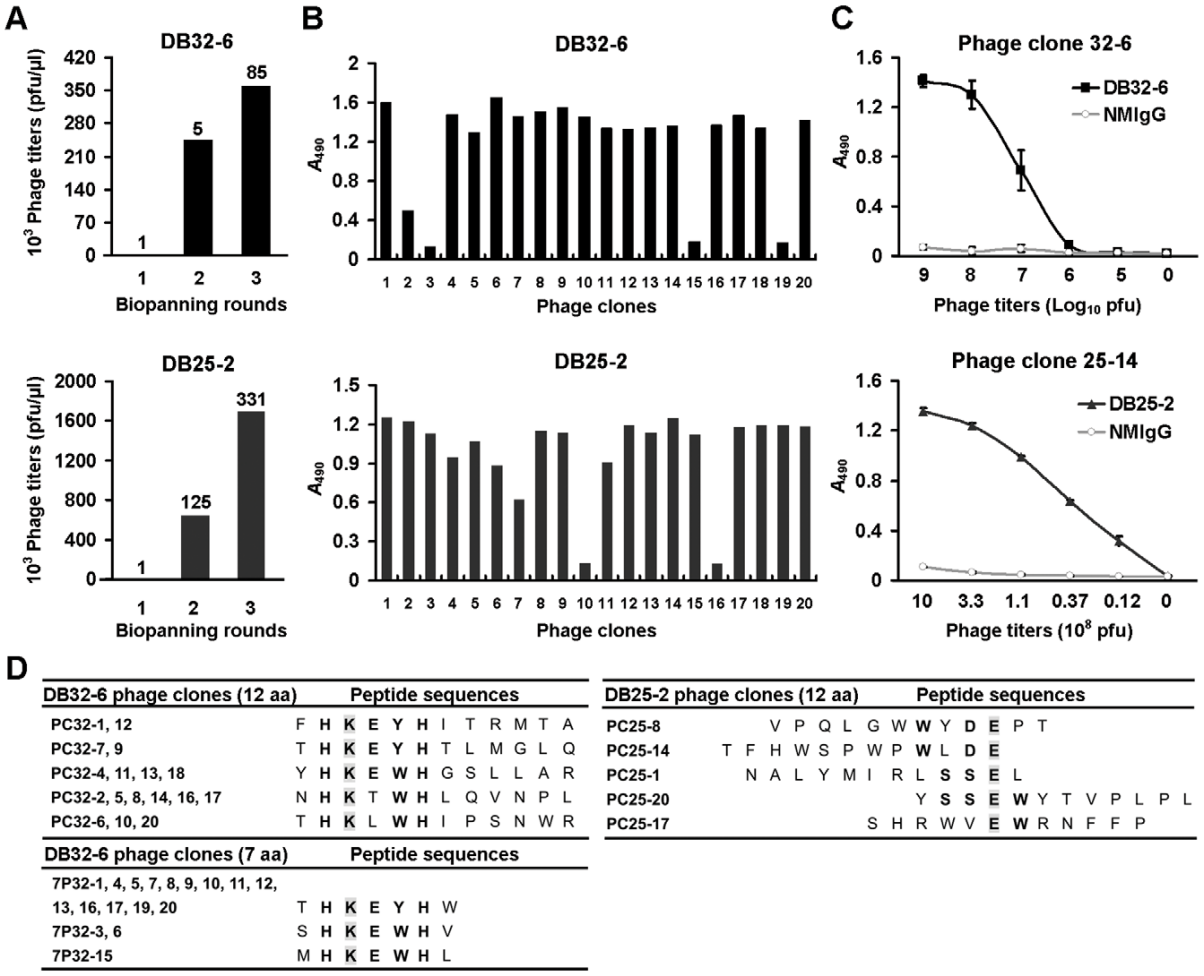
Screening phage-displayed peptide library with neutralizing mAbs DB32-6 and DB25-2. (A) After 3 rounds of biopanning, phage titers were increased to 85-fold (DB32-6) and 331-fold (DB25-2), respectively. (B) Immunopositive phage clones selected by DB32-6 and DB25-2 were identified by ELISA. (C) ELISA reactivity of selected phage clones with DB32-6 and DB25-2. NMIgG was used as a negative control. (D) Alignment of phage-displayed peptide sequences selected by DB32-6 and DB25-2. Consensus motifs are indicated in boldface type. The consensus amino acids of DB32-6 and DB25-2 epitopes are marked in gray.

Epitopes recognized by neutralizing antibodies have been identified in all three domains of the E protein [42]–[44]. To find out more about the epitopes of these neutralizing antibodies, we used phage display [29], [45] to identify the neutralizing epitopes. After three rounds of phage display biopanning, the phage titers were increased to 85-fold (DB32-6) and 331-fold (DB25-2) compared to the phage display biopanning results from the first round (Figure 3A). Individual phage clones from the third round of biopanning were randomly selected. ELISA was performed to determine whether the mAbs could specifically recognize selected phage clones. Of 20 selected phage clones, 17 and 18 clones had significant enhancement of binding activity to DB32-6 and DB25-2, respectively (Figure 3B). The selected phage clones PC32-6 and PC25-14 were specific and dose dependently bound to DB32-6 and DB25-2, respectively. They did not react with control NMIgG (Figure 3C).

The 17 immunopositive phage clones that were highly reactive with DB32-6 were amplified and phage DNA was isolated for DNA sequencing. All of the phage clones displayed 12 amino acid (aa) residues (Figure 3D left). Phage-displayed peptide sequences selected by DB32-6 had the consensus motifs of histidine (H)-lysine (K)-glutamic acid (E)-tryptophan (W)/tyrosine (Y)-histidine (H) (Figure 3D left). Similarly, 17 immunopositive phage clones selected by DB32-6 using phage library displayed 7 amino acid residues, which contained the consensus motif H-K-E-W/Y-H (Figure 3D left). Interestingly, all phage-displayed peptides selected by DB32-6 and DB25-2 contained lysine (K) and glutamic acid (E), respectively (Figure 3D).

**Figure 4:**
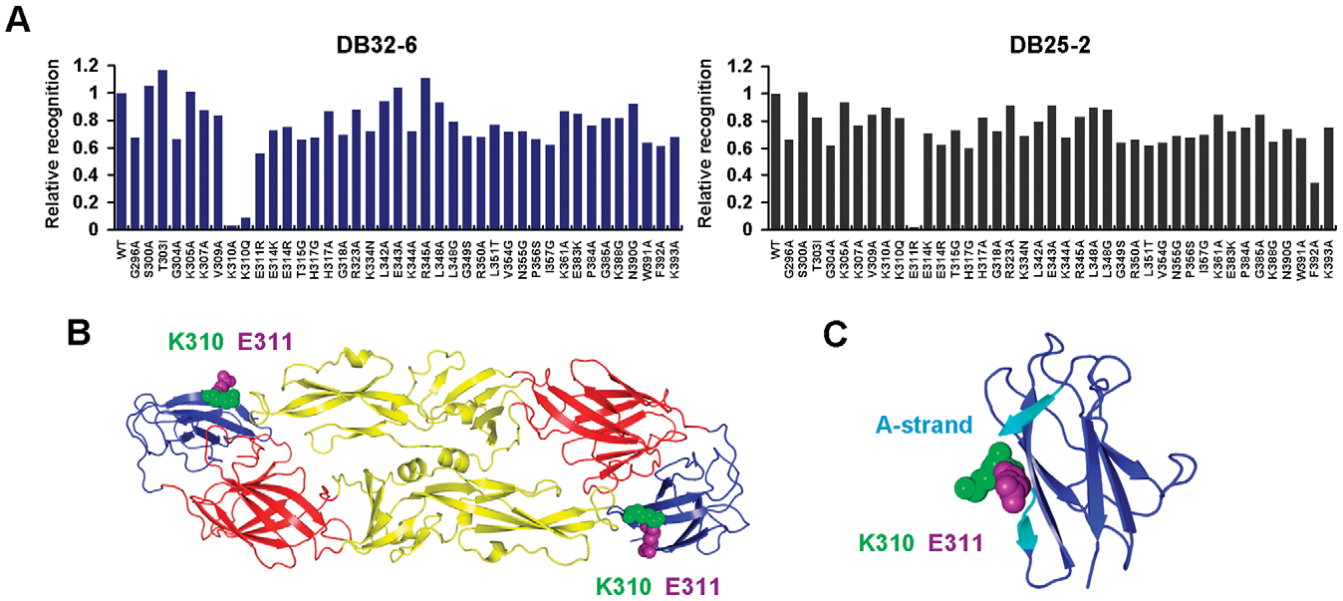
Identification of neutralizing epitopes of mAbs against DENV-2. (A) Various DENV-2 virus-like particle (VLP) mutants were expressed in BHK-21 cells. After fixation and permeabilization, the cells were incubated with DB32-6 and DB25-2. Binding activity was assessed by flow cytometry. The fluorescence intensities were quantified to determine the relative recognition. Substitutions of K310 and E311 led to a significant loss of binding activity of neutralizing mAbs DB32-6 and DB25-2, respectively. Data shown are one representative experiment out of three independent experiments. (B) Location of neutralizing epitopes on DENV-2 E protein. Structure of DENV-2 E protein (1OAN, Protein Data Bank) is shown as a ribbon diagram. E protein consisted of three domains designated DI (red), DII (yellow) and DIII (blue). The native E protein is a homodimer on the surface of the virus. The serotype-specific neutralizing epitopes located in E-DIII were K310 (green) and E311 (purple). (C) Ribbon diagram of neutralizing epitopes K310 (green) and E311 (purple) in A-strand (cyan) of E-DIII.

To further confirm the neutralizing epitopes, we developed various E protein epitope-specific variants VLPs and screened loss-of-binding VLP mutants for identification of critical recognition residues. Using this strategy, we found that DB32-6 lost its VLP binding activity when the residue K310 in the A-strand of E-DIII was changed to alanine (K310A) or glutamine (K310Q) (Figure 4A left). Similarly, DB25-2 lost its VLP binding activity when E311 was changed to arginine (E311R) in the A-strand of E-DIII (Figure 4A right). Both the critical recognition residues K310 and E311 were located in the A-strand of E-DIII (Figures 4B and 4C). We found that mAb 3H5 recognized residues K305, E383 and P384 (Figure S4), as previously reported [17], [20]. Notably, even the adjacent residues (K310 and E311) induced antibodies with different levels of neutralizing activity. By comparing the amino acid sequences of E proteins from representing genotypes of DENV-2 (Table S1), we found residues K310 and E311 in E-DIII of the different genotypes (Southeast Asian, West African and American) (Figure S5). Our data further showed epitopes in the A-strand of E-DIII were important for inducing neutralizing antibodies.

### Development of humanized DB32-6 mAbs

**Figure 5:**
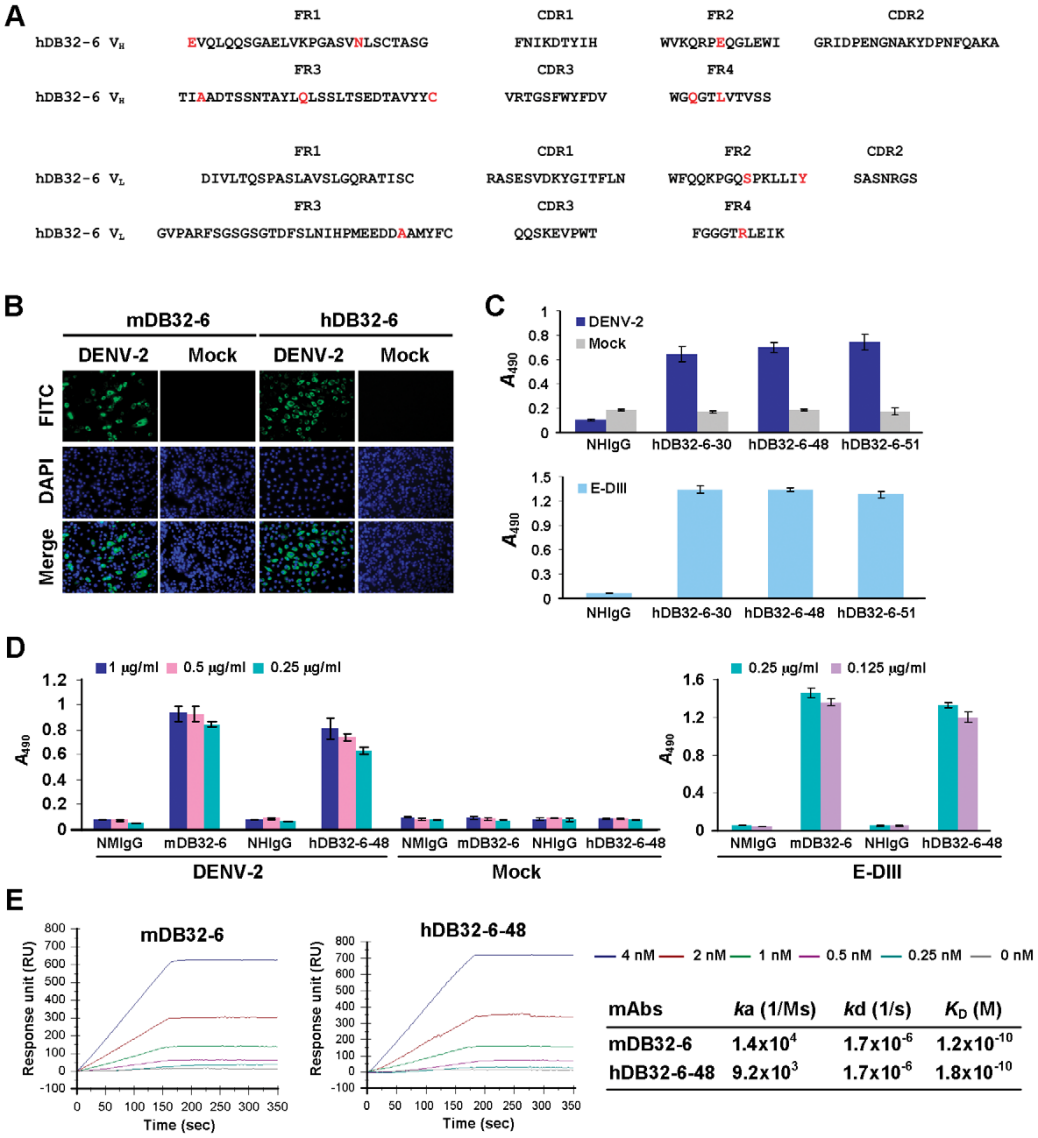
Construction and characterization of humanized DB32-6 mAb. (A) Amino acid sequences of humanized DB32-6 (hDB32-6). FR, framework region; CDR, complementarity determining region. Red residues represent the different amino acids from murine DB32-6 (mDB32-6). (B) mDB32-6 and hDB32-6 mAbs recognized DENV-2-infected BHK-21 cells by IFA. Cells were counterstained with DAPI (blue) and observed at 400× magnification. (C) Binding activity of hDB32-6 mAbs. Three stable clones of hDB32-6 (hDB32-6-30, hDB32-6-48 and hDB32-6-51) recognized DENV-2-infected C6/36 cells and recombinant E-DIII of DENV-2 by ELISA. (D) Various concentrations of mDB32-6 and hDB32-6-48 mAbs were reactive to DENV-2 and recombinant E-DIII of DENV-2 but not to mock control. NMIgG and NHIgG were used as negative controls. (E) Binding affinities of mDB32-6 and hDB32-6-48 to E-DIII of DENV-2. mAbs affinity analysis was performed by surface plasmon resonance (SPR). Binding affinity was tested at the mAb concentrations ranging 0 to 4 nM. Binding curves and kinetic parameters are shown.

Murine mAbs have been shown to have limited clinical use because of their short serum half-life, inability to trigger human effector functions and the production of human anti-murine antibodies (HAMA) response [46]. mAbs have been humanized by grafting their CDRs onto the V_H_ and V_L_ FRs of human Ig molecules [47]. DB32-6 was the most potent mAb against DENV-2 and showed potential as a therapeutic antibody. To develop humanized mAbs, we sequenced V_H_ and V_L_ segment of the neutralizing mAbs from hybridoma cell lines. The CDRs of DB32-6 were grafted onto human IgG1 backbone to create humanized DB32-6 (hDB32-6) (Figure 5A). The hDB32-6 was expressed in CHO-K1 cells and purified from culture supernatants. Both hDB32-6 and mDB32-6 were able to against DENV-2 (Figure 5B). The hDB32-6 maintained the specificity of murine DB32-6 (mDB32-6). Furthermore, we established stable clones of hDB32-6. After selection, mAbs hDB32-6-30, hDB32-6-48 and hDB32-6-51 were found to have highly binding activity (Figure 5C). Comparing to these mAbs, we found hDB32-6-48 to have the highest production rate in cells. mAb hDB32-6-48 was dose-dependent against DENV-2 and E-DIII (Figure 5D). The affinity was analyzed by surface plasmon resonance. The mDB32-6 and hDB32-6-48 bound to E-DIII of DENV-2 with a similar affinity (0.12 nM and 0.18 nM, respectively) (Figure 5E). The results revealed that hDB32-6 maintained the same binding affinity to the E protein as mDB32-6.

### mAb hDB32-6 protected mice from DENV-2-induced mortality

**Figure 6:**
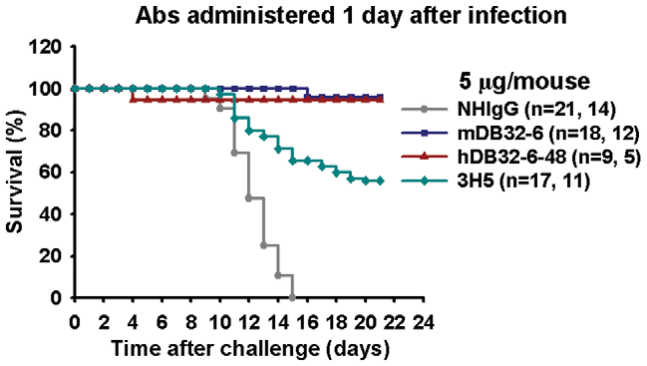
mAbs, mDB32-6 and hDB32-6-48, protected against DENV-2-induced mice mortality. Two-day-old suckling mice (ICR strain) were injected intracranially (i.c.) with 1×10^4^ pfu of DENV-2 (16681). After 1 day of infection, 5 µg of mAb were passively injected into mice through an i.c. route. Log rank test was used to determine significant differences in survival rate, and the mAbs with neutralizing activity were compared to the control group treated with NHIgG, *P*<0.001.

We established a suckling mice model to determine the protective activity of mDB32-6 and hDB32-6. To evaluate therapeutic effect of mAbs, we administered 5 µg of mAb at day one after 1×10^4^ pfu (25-fold LD_50_) of DENV-2 (16681) infection. Through 21 days of observation, groups treated with mDB32-6, hDB32-6-48 and 3H5 mAbs were found to have survival rates of 96%, 94% and 56%, respectively (Figure 6). However, none of the mice in control antibody normal human IgG (NHIgG)-treated group survived (Figures 6). These results demonstrate that both mDB32-6 and hDB32-6 have excellent neutralizing activity against DENV-2.

### mAb hDB32-6 variant eliminate ADE phenomenon

**Figure 7:**
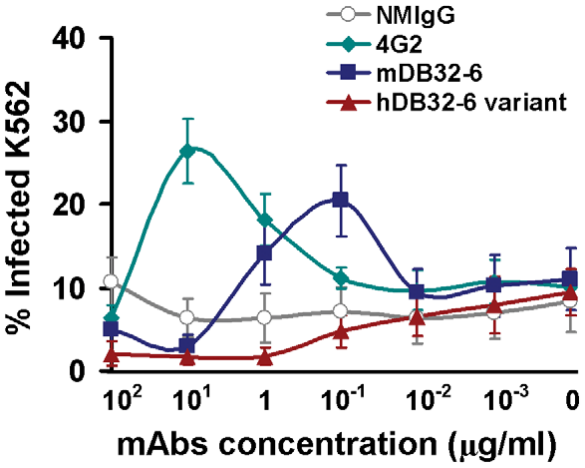
Antibody-mediated enhancement of DENV-2 infection by mAbs. Serial dilutions of NMIgG, 4G2, mDB32-6 and hDB32-6 variant were incubated with DENV-2 (16681) at MOI of 1 at 4°C for 1 h before they were added to K562 cells. After 2 days infection, cells were fixed, permeabilized, and stained with mAb DB42-3, and the percentage of cells infected with DENV-2 was detected by flow cytometry.

When developing the antibody-based therapy, ADE phenomenon is a major cause for concern in dengue pathogenesis because it might enhance DENV infection. Modification of Fc structure in an antibody can prevent Fcγ receptors binding and lead to eliminate ADE [38], [39], [48]. We generated a variant of humanized DB32-6 (hDB32-6 variant) to prevent Fcγ receptors binding while maintaining DENV neutralizing capability without enhancing infection (Figure 7). The hDB32-6 variant retained the same neutralizing activity as unmodified mAb mDB32-6 at high concentrations (100 µg/ml and 10 µg/ml) but was completely devoid of enhancing activity at low concentrations (1 µg/ml and 0.1 µg/ml) (Figure 7). The hDB32-6 variant eliminated the ADE phenomenon and holds great potential for being developed into therapeutic antibodies for the prevention and treatment of DENV-2 infection.

## Discussion

mAbs of DENV have served as powerful research tools for antiviral development and pathological investigations. Here, we newly generated and characterized 17 mAbs with high reactivity against E protein of DENV-2. Several mAbs had potent neutralizing activity. The neutralizing epitopes were identified using a combination of strategies, including phage display, computational structure analysis [49], and high-throughput epitope mapping of VLPs. From these results, the A-strand of E-DIII was found to be important in neutralizing DENV-2 than the lateral ridge of E-DIII. mAb DB32-6 which had the strongest neutralizing activity against various strains of DENV-2 was humanized and modified to abrogate the ADE phenomenon. The mAb DB32-6 was demonstrated to increase the survival rate in two mouse models even after DENV-2 infection.

Based on previous epitope mapping results, several epitopes have been shown to elicit strong neutralizing antibodies against individual flaviviruses that situated in E-DIII [14], [50]. Investigation of neutralizing epitopes on the E proteins may provide the framework for a detailed understanding of both specific mechanisms of the viral infection as well as the identification of the specific DENV domain that attaches to a cellular receptor. Phage display is useful in the identification of B-cell epitopes, including linear [32], [51] and conformational epitopes [29], [45]. However, these epitopes need to further elucidation using other methods. Combining different strategies provided a fast and reliable evidence for identifying epitopes (Figures 3 and 4). To date, few mAbs possess better neutralizing activity than 3H5, which has been shown to bind to residues K305, E383 and P384 at the lateral ridge of E-DIII [17], [20]. DB32-6 had higher neutralizing activity than 3H5. Neutralizing epitope of DB32-6 was mapped on K310 residue in A-strand of E-DIII (Figure 4). Neutralizing epitope of another mAb DB25-2 was mapped on E311 residue in A-strand of E-DIII, too (Figure 4). These serotype-specific neutralizing epitopes located in the A-strand of E-DIII induced stronger neutralizing activity than those located on the lateral ridge of E-DIII. We aligned different DENV-2 genotypes and found that the K310 and E311 were frequently observed in DENV-2 (Figure S5). The K310 may be important to DENV-2. Thus by binding DB32-6 to K310, it lead to dramatic neutralized DENV-2. To determine whether DB32-6 can neutralize diverse genotypes of DENV-2 is a critical step in evaluating the potential of therapeutic development in the future.

Previous studies have shown that the strongly neutralizing mAb, subcomplex-specific 1A1D-2 and cross-reactive 9F12 recognized residues at K305, K307 and K310 in A-strand [15], [17]. Our mAb DB32-6 is a serotype-specific neutralizing mAb that recognized residue K310 but not residues K305 or K307. Although K310 is considered as a subcomplex-specific epitope, DB32-6 is a serotype-specific mAb. There may be other regions that affect the binding of DB32-6 to DENV-2. We found that by mutating residue I312, DB32-6's binding activity was reduced by 50% (data not shown). Residue I312 may be a minor epitope of DB32-6. Moreover, 1A1D-2 is a temperature dependent mAb due to its needs for dynamic motion on the virion surface to neutralize virus [16]. Different from 1A1D-2, DB32-6 is temperature independent. When DB32-6 was incubated with DENV at 4°C, it still exhibited significant neutralizing activity (Figures 2 and 6). As expected, when incubating the DENV and DB32-6 at 37°C, DB32-6 showed better efficacy than it did at 4°C (data not shown). The residue K310 on the surface of DENV-2 may be accessible to DB32-6 binding. Additionally, DB32-6 had high binding affinity (0.12–0.18 nM) to DENV-2. Based on the above finding, the residue K310 induce serotype-specific mAbs and is crucial in the neutralization of virus infectivity.

Antibodies to E-DI-II tend to be more cross-reactive and less potent in neutralization of dengue infection [39]. However, there are fewer antibody concentrations capable of recognizing E-DIII than there are that recognize E-DI-II in dengue patients [20], [39]. Wahala et al. studied the human immune sera of DENV infection and found the E-DIII binding antibodies to play a minor role in DENV neutralization, similar to West Nile virus-infected human [52], [53]. The mAbs that bind to E-DIII expresses potent neutralizing activity, but only a few of them exist in serum of the patients infected with DENV or WNV. Combining the information from both mice and human mAbs studies of DENV infection is critical to understanding the complex mechanism behind the humoral immunity following natural DENV infection. According to one previous study, the immunoglobulin populations recognizing residues K310, E311 and P364 in dengue fever patients were much larger in IgM than in IgG [20]. The strong neutralizing IgG made up a small proportion of the antibody in dengue patients. de Alwis et al. has conducted an in-depth analysis of the human mAbs derived from memory B-cells of patients infected with primary DENV infections [54]. After the epitope mapping of anti-DENV-2 human mAbs, the strong neutralizing mAb 10.16 was mapped to K305, K310 and E311 in the A-strand. Together, the finding above suggest that the highly protective epitopes K310 and E311 in mouse play a role in humans as well.

We also identified several E-DI-II specific mAbs with high to no neutralizing activity. Serotype-specific mAbs (DB2-3 and DB23-3) with potent neutralizing activity were found to recognize E-DI-II of DENV-2 (Figure 2 and Table 1). Some studies have identified highly neutralizing and protective antibodies against JEV and DENV located in E-DI [55], [56] Currently, we are in the process of identifying the neutralizing epitopes of DB2-3 and DB23-3. mAbs that broadly cross-react with other flaviviruses are in E-DII near the fusion loop, which is immunodominant antigenic [20], [34], [42]. Binding an antibody to DENV can change the rearrangement of the E protein, which may neutralize or enhance viral infection [16], [57]. The high or no neutralizing activity of our mAbs can be used help identify neutralizing or immunopathogenic epitopes in the E protein. Studies that explore the mAbs mediated neutralization mechanism and mAbs dependent enhancement are currently underway.

The mouse models for dengue infection developed to date do not represented the entirety of the pathogenesis of human dengue infection [58]. Developing of mouse models to studying its pathogenesis is important but challenging. We used two models, suckling mice protection assay and *Stat1*-deficient *(Stat1^−/−^)* mouse model with different DENV-2 strains through intracerebral or intraperitoneal inoculation to evaluate the neutralizing activity of DB32-6 mAb (Figures 2 and 6). Our findings suggested that mAb DB32-6 might effectively block virus entry. However, disease manifestation of suckling mice is not relevant to dengue disease in humans since DENV infections in humans rarely involve the nervous system [58]. The *Stat1*-deficient mice are genetically mutated and not immunocompetent, hence they are not representative of the wild types' immune response to DENV. However, their survival rates might reflect the therapeutic potential of these mAbs. The results from these mouse models showed that the therapeutic potential of this newly generated mAb DB32-6 is worth further investigation.

In the absence of an effective dengue vaccine, neutralizing antibodies can be used as a passive immunotherapeutic strategy for treating dengue. Previous studies of humanized antibodies against DENV were derived from two chimpanzee Fab fragments: humanized IgG1 1A5 cross-neutralizing DENV-1 and DENV2 and humanized IgG1 5H2 specific against DENV-4 [42], [48], [56]. Our newly generated hDB32-6 was derived from murine mAb. However, when developing antibody-based therapy, ADE phenomenon is a major concern. Modification of Fc structure in an antibody can prevent Fcγ receptors binding and inhibit ADE (Figure 7) [39], [48].

Our studies show that the serotype-specific mAbs targeting the A-strand of E-DIII could serve as a dramatic neutralization determinant. Through testing in different mouse models, we have successfully generated a mAb hDB32-6 variant with high therapeutic potential against diverse DENV-2 strains without inducing ADE. Such an antibody-based therapy may help control severe dengue in the future.

## Supporting Information

Figure S1 **Specificity of mAbs against DENV.** mAbs recognized DENV-2 (16681) infected BHK-21 cells by immunofluorescence assay. BHK-21 cells were infected at a multiplicity of infection (MOI) of 0.5 with DENV-2. At 48 hours post-inoculation, antigen was detected by staining with mAbs to DENV-2, followed by staining with FITC conjugated goat anti-mouse IgG antibodies (green). Cells were counterstained with DAPI (blue) and examined under fluorescence microscopy (Zeiss). Cells images were acquired at 400× magnification.
(DOC)

Figure S2 **Expression of DENV-2 proteins in BHK-21 cells.** BHK-21 cells were transfected with plasmids of DENV-2 C, prM, prM-E, E, NS1, NS2A, NS2B, NS2B-3, NS3, NS4A, NS4B and NS5. After 48 hours, antigen was detected by mAbs, followed by staining with FITC conjugated goat anti-mouse IgG antibodies (green). Cells were counterstained with DAPI (blue) and examined under fluorescence microscopy (Zeiss). Cells images were acquired at 400× magnification.
(DOC)

Figure S3 **DB32-6-mediated neutralization of different DENV-2 genotypes infection.** Serial dilutions of DB32-6 mAb were incubated with DENV-2 (16681, NGC, PL046 and Malaysia 07587) at MOI of 0.5 at 4°C for 1 hour before they were added to BHK-21 cells. After 2 days infection, the percentages of infected cells were assessed by flow cytometry.
(DOC)

Figure S4 **Identification of mAb 3H5 neutralizing epitopes by VLP mutants.** BHK-21 cells expressed various DENV-2 VLP mutants. After fixation and permeabilization, mAbs were incubated with the cells. Binding activity was assessed by flow cytometry. The fluorescence intensities were quantified to determine the relative recognition, calculated as [intensity of mutant VLP/intensity of WT VLP] (recognized by a mAb)×[intensity of WT VLP/intensity of mutant VLP] (recognized by mixed mAbs). Data shown are one representative experiment out of three independent experiments.
(DOC)

Figure S5 **Sequence alignment of different DENV-2 genotypes and highlights of the neutralizing epitopes in E-DIII.** The sequence of E-DIII from DENV-2 (strain 16681, Southeast Asian genotype) is aligned with other DENV-2 genotypes including NGC (Southeast Asian), PL046 (Southeast Asian), PM33974 (West African) and IQT2913 (American). Black blocks show residues of genotypic variation. The serotype-specific neutralizing epitopes located in E-DIII are K310 (green) and E311 (purple) which are recognized by DB32-6 and DB25-2, respectively.
(DOC)

Table S1 **The database, gene/protein and accession/ID number were mentioned in the text.**
(DOC)
