# Correction: Development of a Humanized Antibody with High Therapeutic Potential against Dengue Virus Type 2

**DOI:** 10.1371/journal.pntd.0012031

**Published:** 2024-03-13

**Authors:** Pi-Chun Li, Mei-Ying Liao, Ping-Chang Cheng, Jian-Jong Liang, I-Ju Liu, Chien-Yu Chiu, Yi-Ling Lin, Gwong-Jen J. Chang, Han-Chung Wu

The DB22-4 panels in Fig 1C [1] were erroneously used to represent the DB22-4 results in Fig 1D. An updated version of Fig 1 is provided here in which the DB22-4 panels in Fig 1D are replaced.

Original images underlying Fig 1C and 1D are provided in S1 and S2 Files.

The original underlying data to support all results in the article and Supporting Information files are available from the corresponding author, except for the original western blot images underlying the DB19-4 panel in Fig 1B, which are no longer available.

The authors apologize for the error in the published article.

**Fig 1:**
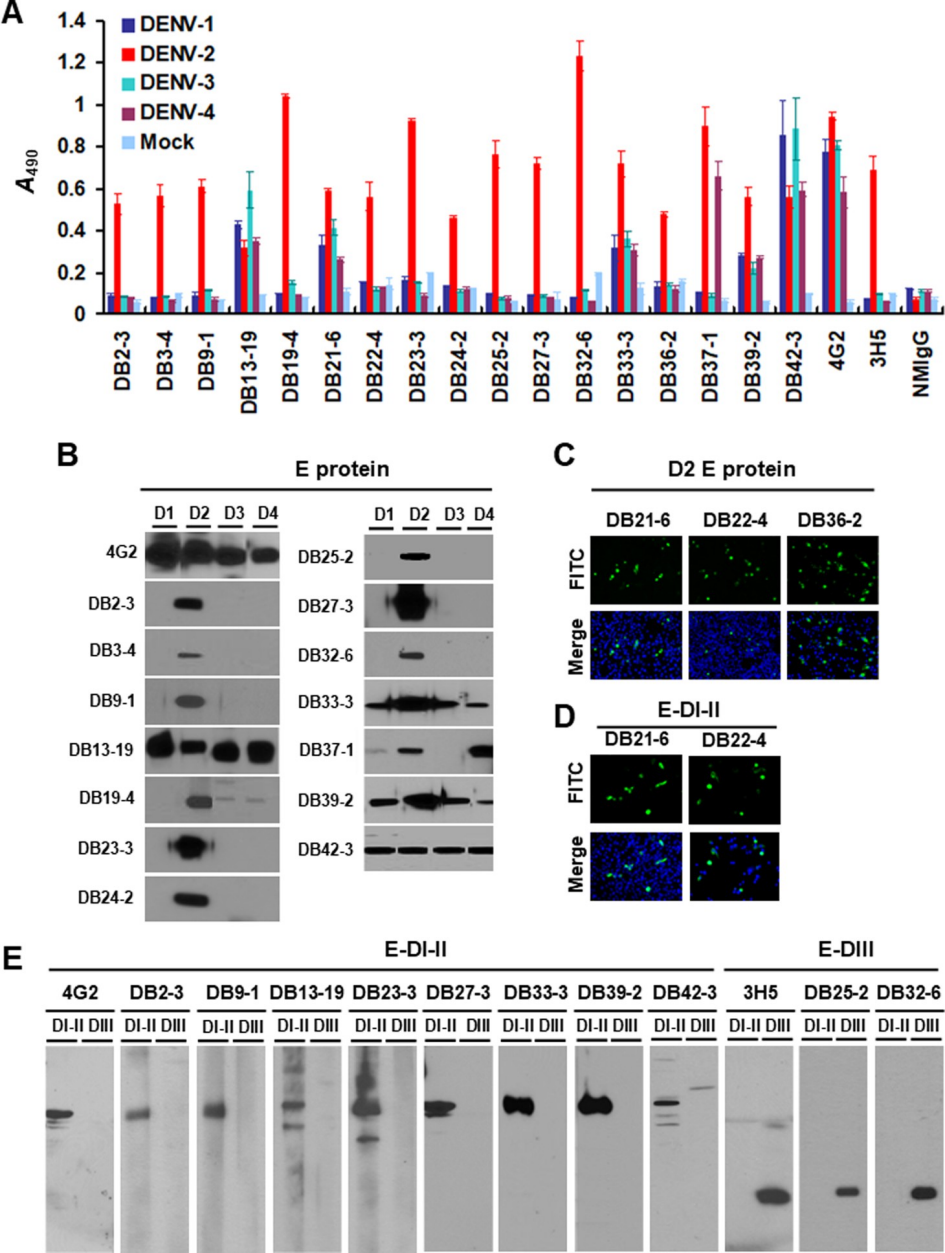
Characterization of mAbs against DENV. A) C6/36 insect cells were infected by DENV-1, -2, -3 and -4 or uninfected (Mock). After fixation and permeabilization, mAbs were incubated with cells and binding was assessed by cellular ELISA. A_490_, optical density at 490 nm. (B) Identification of mAbs by Western blotting. C6/36 cells were infected with DENV-1 to -4 (D1, D2, D3 and D4) as viral antigens. Protein samples were dissolved in native sample buffer and fractionated by 10% SDS-PAGE. mAbs recognized E protein (53 kDa) of DENV. (C and D) mAbs recognized DENV-2 E protein and E-DI-II was determined by IFA, respectively. (E) Dissection of DENV-2 mAbs recognized E-DI-II or E-DIII by Western blot analysis. The DENV-2 recombinant E-DI-II-flag (36 kDa) and E-DIII-flag (17 kDa) fusion proteins were expressed in *Escherichia coli*. Protein extract was dissolved in denatured sample buffer and fractionated on 12% SDS-PAGE. 4G2, a cross-reactive mAb and 3H5, a DENV-2 serotype-specific mAb recognized D2-E-DI-II and D2-E-DIII, respectively. They were used as positive controls.

## Supporting information

S1 File Original images underlying Fig 1C. (ZIP)

S2 File Original images underlying Fig 1D. (PPTX)

## References

[1] Li P-C, Liao M-Y, Cheng P-C, Liang J-J, Liu I-J, Chiu C-Y, et al. (2012) Development of a Humanized Antibody with High Therapeutic Potential against Dengue Virus Type 2. PLoS Negl Trop Dis 6(5): e1636. doi: 10.1371/journal.pntd.0001636 22563515 PMC3341331

