# Supplementary material for: Development of a Humanized Antibody with High Therapeutic Potential against Dengue Virus Type 2
Source: PLoS Negl Trop Dis. 2012 May 1;6(5):e1636. doi: 10.1371/journal.pntd.0001636 (PMC3341331; doi:10.1371/journal.pntd.0001636)
Supplement: Figure S1 — Specificity of mAbs against DENV. mAbs recognized DENV-2 (16681) infected BHK-21 cells by immunofluorescence assay. BHK-21 cells were infected at a multiplicity of infection (MOI) of 0.5 with DENV-2. At 48 hours post-inoculation, antigen was detected by staining with mAbs to DENV-2, followed by staining with FITC conjugated goat anti-mouse IgG antibodies (green). Cells were counterstained with DAPI (blue) and examined under fluorescence microscopy (Zeiss). Cells images were acquired at 400× magnification. (DOC) [file pntd.0001636.s001.doc]

**
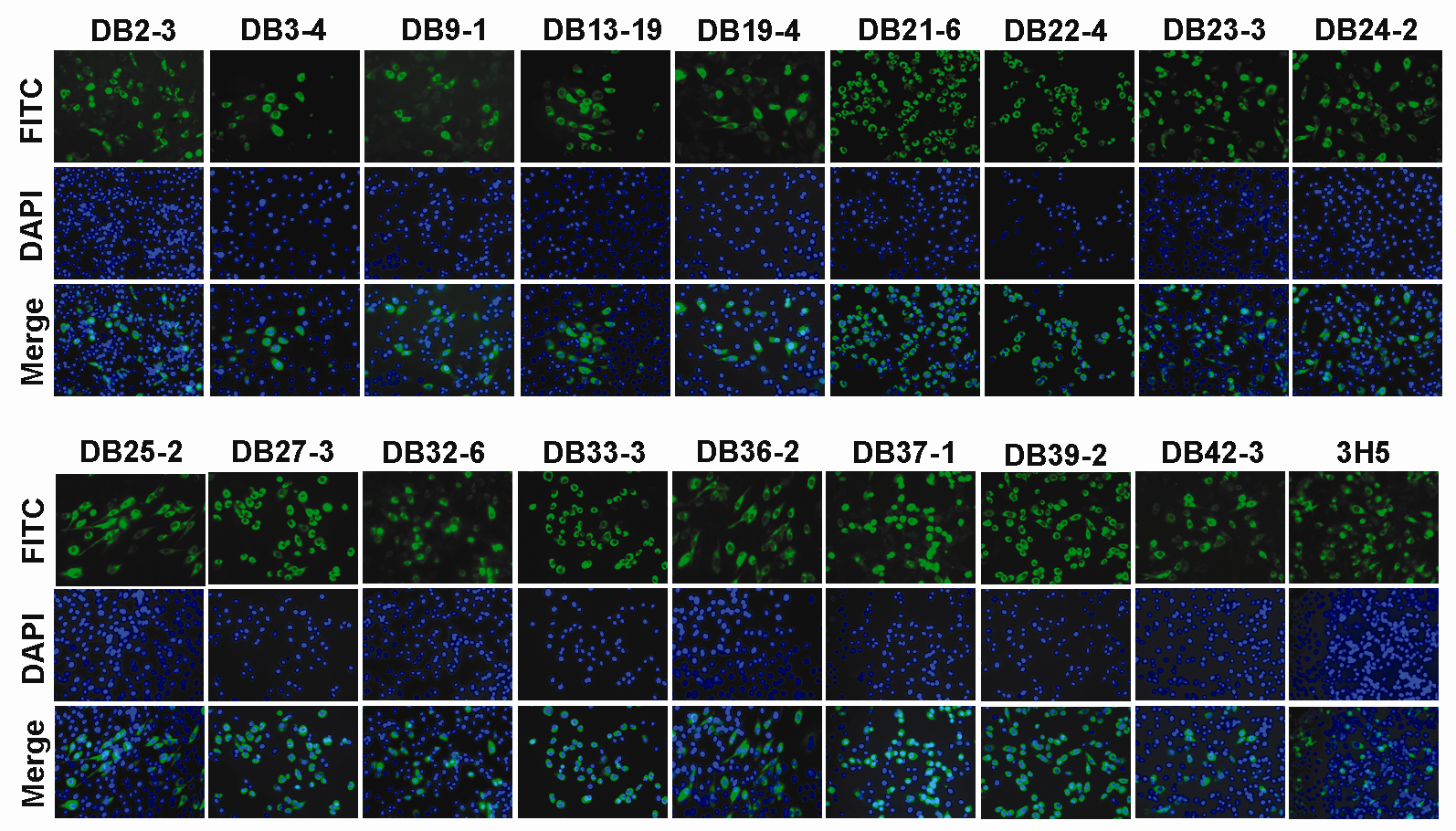
**

**Figure S1. Specificity of mAbs against DENV.** mAbs recognized DENV-2 (16681) infected BHK-21 cells by immunofluorescence assay. BHK-21 cells were infected at a multiplicity of infection (MOI) of 0.5 with DENV-2. At 48 hours post-inoculation, antigen was detected by staining with mAbs to DENV-2, followed by staining with FITC conjugated goat anti-mouse IgG antibodies (green). Cells were counterstained with DAPI (blue) and examined under fluorescence microscopy (Zeiss). Cells images were acquired at 400× magnification.
