# Supplementary material for: Development of a Humanized Antibody with High Therapeutic Potential against Dengue Virus Type 2
Source: PLoS Negl Trop Dis. 2012 May 1;6(5):e1636. doi: 10.1371/journal.pntd.0001636 (PMC3341331; doi:10.1371/journal.pntd.0001636)
Supplement: Figure S2 — Expression of DENV-2 proteins in BHK-21 cells. BHK-21 cells were transfected with plasmids of DENV-2 C, prM, prM-E, E, NS1, NS2A, NS2B, NS2B-3, NS3, NS4A, NS4B and NS5. After 48 hours, antigen was detected by mAbs, followed by staining with FITC conjugated goat anti-mouse IgG antibodies (green). Cells were counterstained with DAPI (blue) and examined under fluorescence microscopy (Zeiss). Cells images were acquired at 400× magnification. (DOC) [file pntd.0001636.s002.doc]

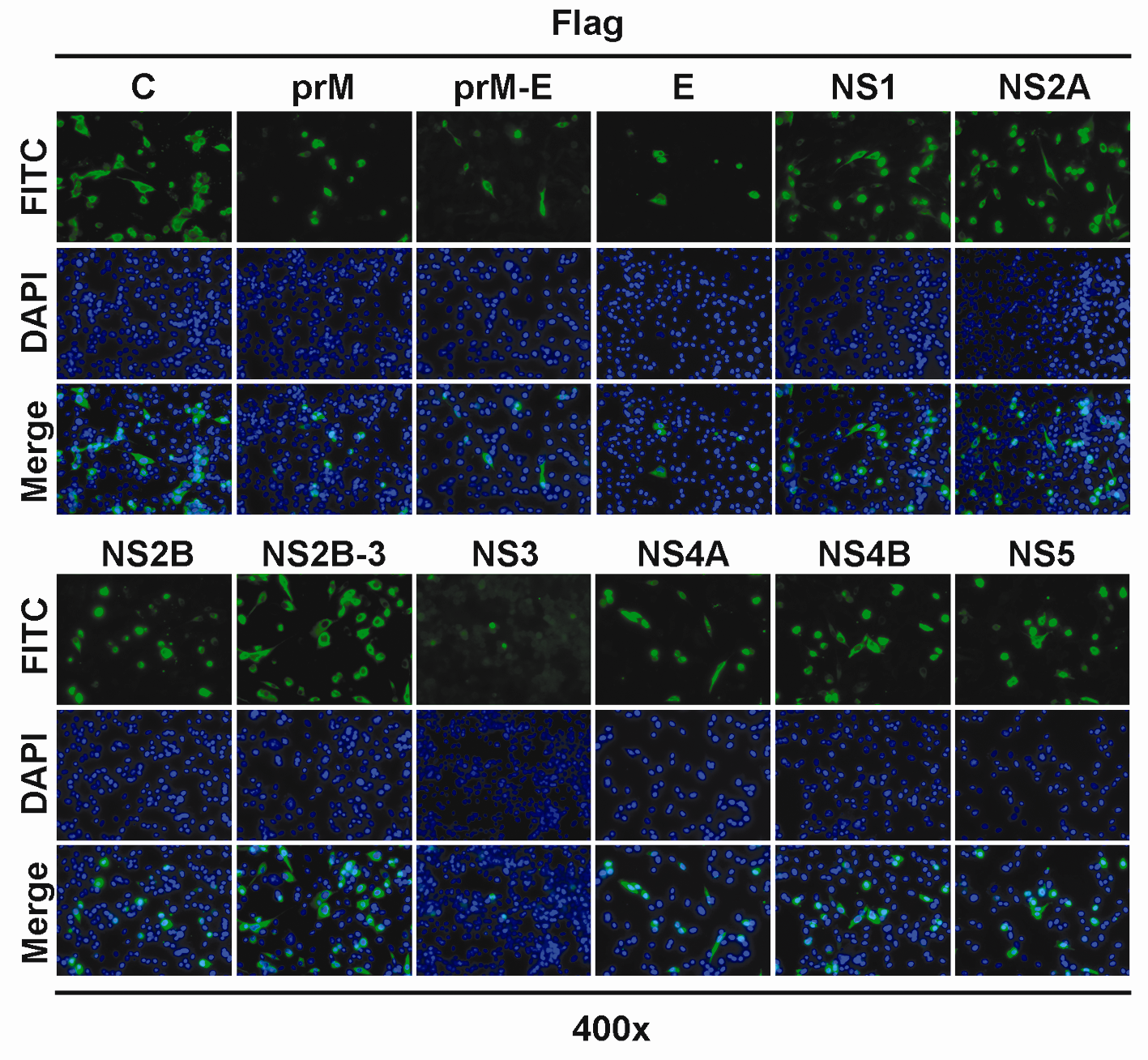


**Figure S2. Expression of DENV-2 proteins in BHK-21 cells.** BHK-21 cells were transfected with plasmids of DENV-2 C, prM, prM-E, E, NS1, NS2A, NS2B, NS2B-3, NS3, NS4A, NS4B and NS5. After 48 hours, antigen was detected by mAbs, followed by staining with FITC conjugated goat anti-mouse IgG antibodies (green). Cells were counterstained with DAPI (blue) and examined under fluorescence microscopy (Zeiss). Cells images were acquired at 400× magnification.
