# Supplementary material for: Development of a Humanized Antibody with High Therapeutic Potential against Dengue Virus Type 2
Source: PLoS Negl Trop Dis. 2012 May 1;6(5):e1636. doi: 10.1371/journal.pntd.0001636 (PMC3341331; doi:10.1371/journal.pntd.0001636)
Supplement: Figure S3 — DB32-6-mediated neutralization of different DENV-2 genotypes infection. Serial dilutions of DB32-6 mAb were incubated with DENV-2 (16681, NGC, PL046 and Malaysia 07587) at MOI of 0.5 at 4°C for 1 hour before they were added to BHK-21 cells. After 2 days infection, the percentages of infected cells were assessed by flow cytometry. (DOC) [file pntd.0001636.s003.doc]

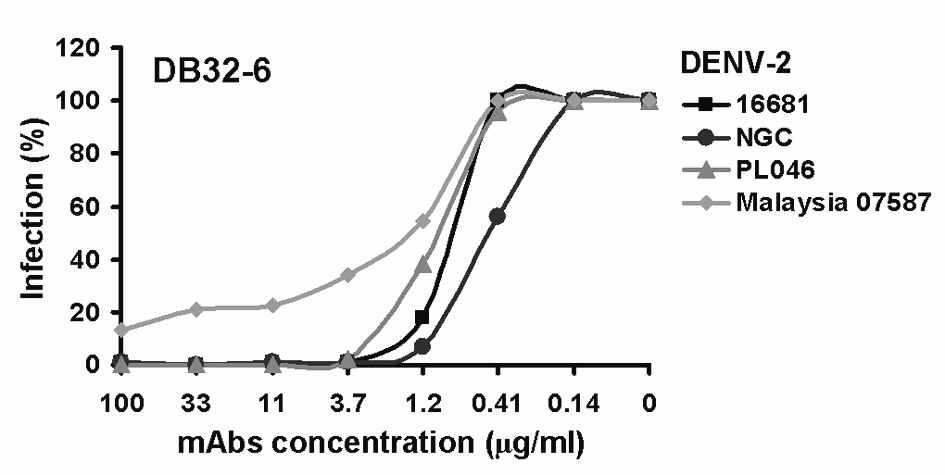


**Figure S3. DB32-6-mediated neutralization of different DENV-2 strains infection.** Serial dilutions of DB32-6 were incubated with DENV-2 (16681, NGC, PL046 and Malaysia 07587) at multiplicity of infection (MOI) of 0.5 at 4℃ for 1 hour before they were added to BHK-21 cells. After 2 days infection, the percentages of infected cells were assessed by flow cytometry.
