# Supplementary material for: Development of a Humanized Antibody with High Therapeutic Potential against Dengue Virus Type 2
Source: PLoS Negl Trop Dis. 2012 May 1;6(5):e1636. doi: 10.1371/journal.pntd.0001636 (PMC3341331; doi:10.1371/journal.pntd.0001636)
Supplement: Figure S4 — Identification of mAb 3H5 neutralizing epitopes by VLP mutants. BHK-21 cells expressed various DENV-2 VLP mutants. After fixation and permeabilization, mAbs were incubated with the cells. Binding activity was assessed by flow cytometry. The fluorescence intensities were quantified to determine the relative recognition, calculated as [intensity of mutant VLP/intensity of WT VLP] (recognized by a mAb)×[intensity of WT VLP/intensity of mutant VLP] (recognized by mixed mAbs). Data shown are one representative experiment out of three independent experiments. (DOC) [file pntd.0001636.s004.doc]

**
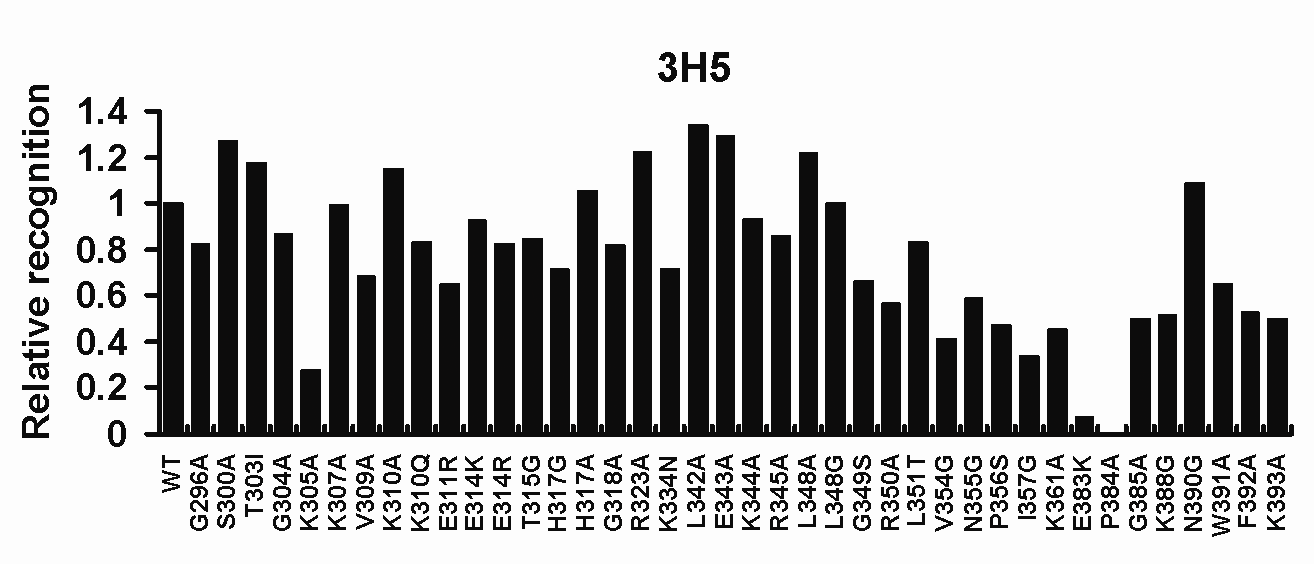
**

**Figure S4. Identification of mAb 3H5 neutralizing epitopes by VLP mutants.** BHK-21 cells expressed various DENV-2 VLP mutants. After fixation and permeabilization, mAbs were incubated with the cells. Binding activity was assessed by flow cytometry. The fluorescence intensities were quantified to determine the relative recognition, calculated as [intensity of mutant VLP /intensity of WT VLP] (recognized by a mAb 3H5) × [intensity of WT VLP /intensity of mutant VLP] (recognized by mixed mAbs). Data shown are one representative experiment out of three independent experiments.
