# Supplementary material for: Development of a Humanized Antibody with High Therapeutic Potential against Dengue Virus Type 2
Source: PLoS Negl Trop Dis. 2012 May 1;6(5):e1636. doi: 10.1371/journal.pntd.0001636 (PMC3341331; doi:10.1371/journal.pntd.0001636)
Supplement: Figure S5 — Sequence alignment of different DENV-2 genotypes and highlights of the neutralizing epitopes in E-DIII. The sequence of E-DIII from DENV-2 (strain 16681, Southeast Asian genotype) is aligned with other DENV-2 genotypes including NGC (Southeast Asian), PL046 (Southeast Asian), PM33974 (West African) and IQT2913 (American). Black blocks show residues of genotypic variation. The serotype-specific neutralizing epitopes located in E-DIII are K310 (green) and E311 (purple) which are recognized by DB32-6 and DB25-2, respectively. (DOC) [file pntd.0001636.s005.doc]

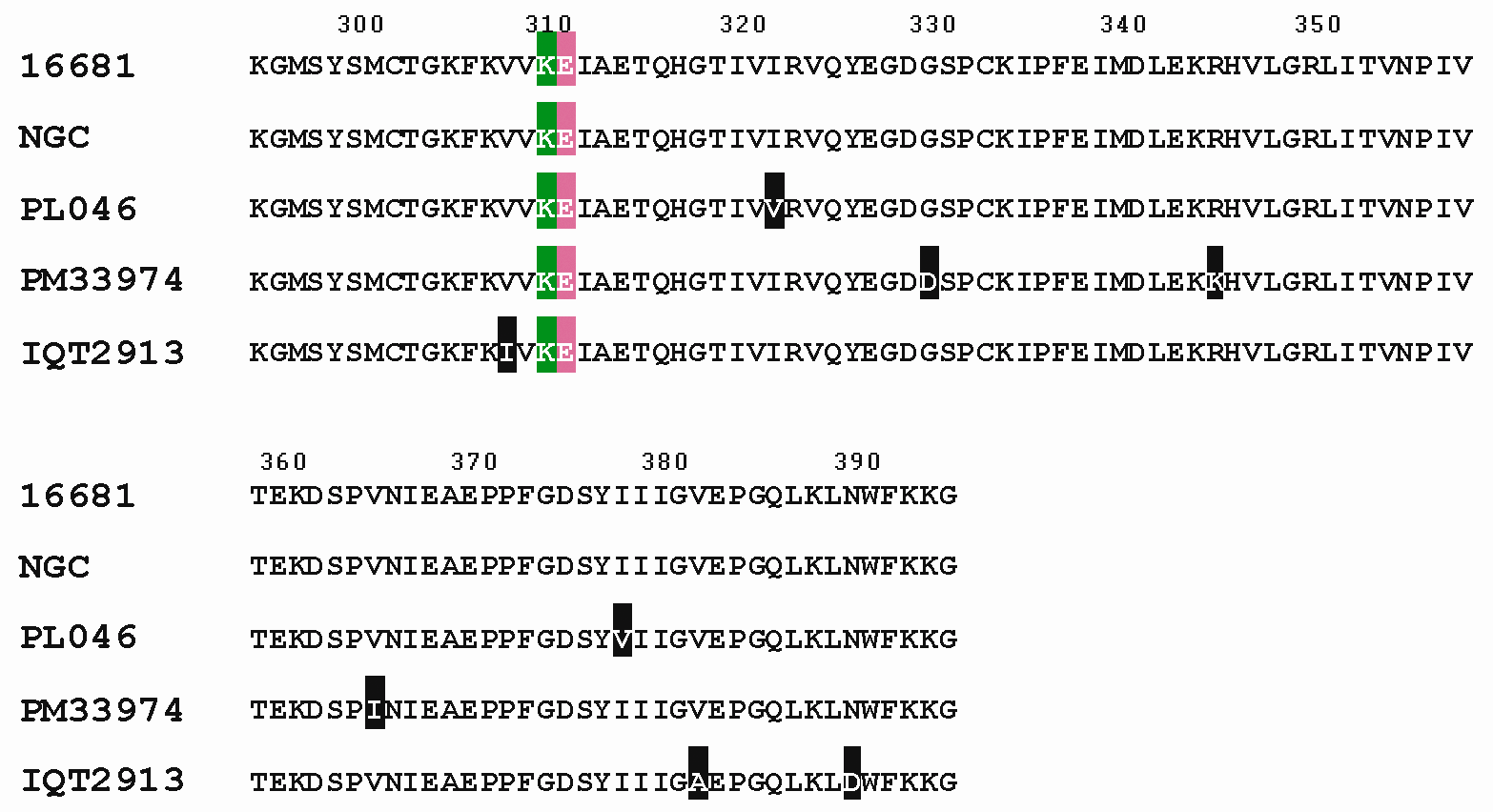


**Figure S5. Sequence alignment of different DENV-2 genotypes and highlights of the neutralizing epitopes in E-DIII.** The sequence of E-DIII from DENV-2 (strain 16681, Southeast Asian genotype) is aligned with other DENV-2 genotypes including NGC (Southeast Asian), PL046 (Southeast Asian), PM33974 (West African) and IQT2913 (American). Black blocks show residues of genotypic variation. The serotype-specific neutralizing epitopes located in E-DIII are K310 (green) and E311 (purple) which are recognized by DB32-6 and DB25-2, respectively.
