# Supplementary material for: Development of a Humanized Antibody with High Therapeutic Potential against Dengue Virus Type 2
Source: PLoS Negl Trop Dis. 2012 May 1;6(5):e1636. doi: 10.1371/journal.pntd.0001636 (PMC3341331; doi:10.1371/journal.pntd.0001636)
Supplement: Table S1 — The database, gene/protein and accession/ID number were mentioned in the text. (DOC) [file pntd.0001636.s006.doc]

**Table S1. The database, gene/protein and accession/ID number were mentioned in the text.**

| **Database** | **Gene/Protein** | **Accession/ID number** |
| --- | --- | --- |
| Protein Data Bank | DENV-2 E protein | 1OAN |
| GenBank | DENV-1 Hawaii | AB609588 |
| GenBank | DENV-2 16681 | M84727 |
| GenBank | DENV-2 NGC | M29095 |
| GenBank | DENV-2 PM33974 | EF105378 |
| GenBank | DENV-2 IQT2913 | AF100468 |
| GenBank | DENV-3 H87 | M93130 |
| GenBank | DENV-4 H241 | AY947539 |
