# Supplementary figures and images for: Correction: Development of a Humanized Antibody with High Therapeutic Potential against Dengue Virus Type 2
Source: PLoS Negl Trop Dis. 2024 Mar 13;18(3):e0012031. doi: 10.1371/journal.pntd.0012031 (PMC10936836; doi:10.1371/journal.pntd.0012031)

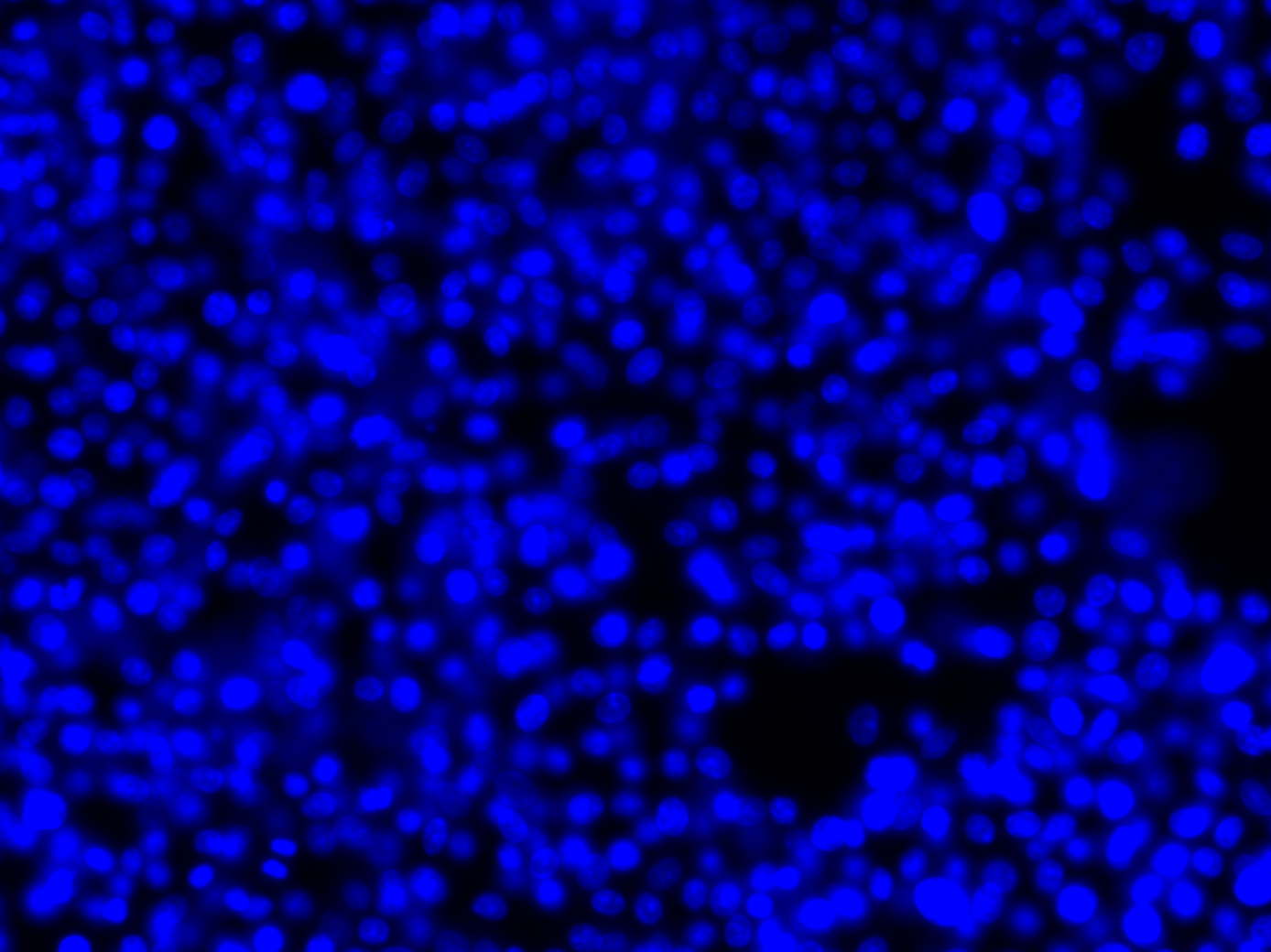

Supplement: S1 File — (ZIP) [file pntd.0012031.s001.zip › DB22-4_E_DAPI.TIF]

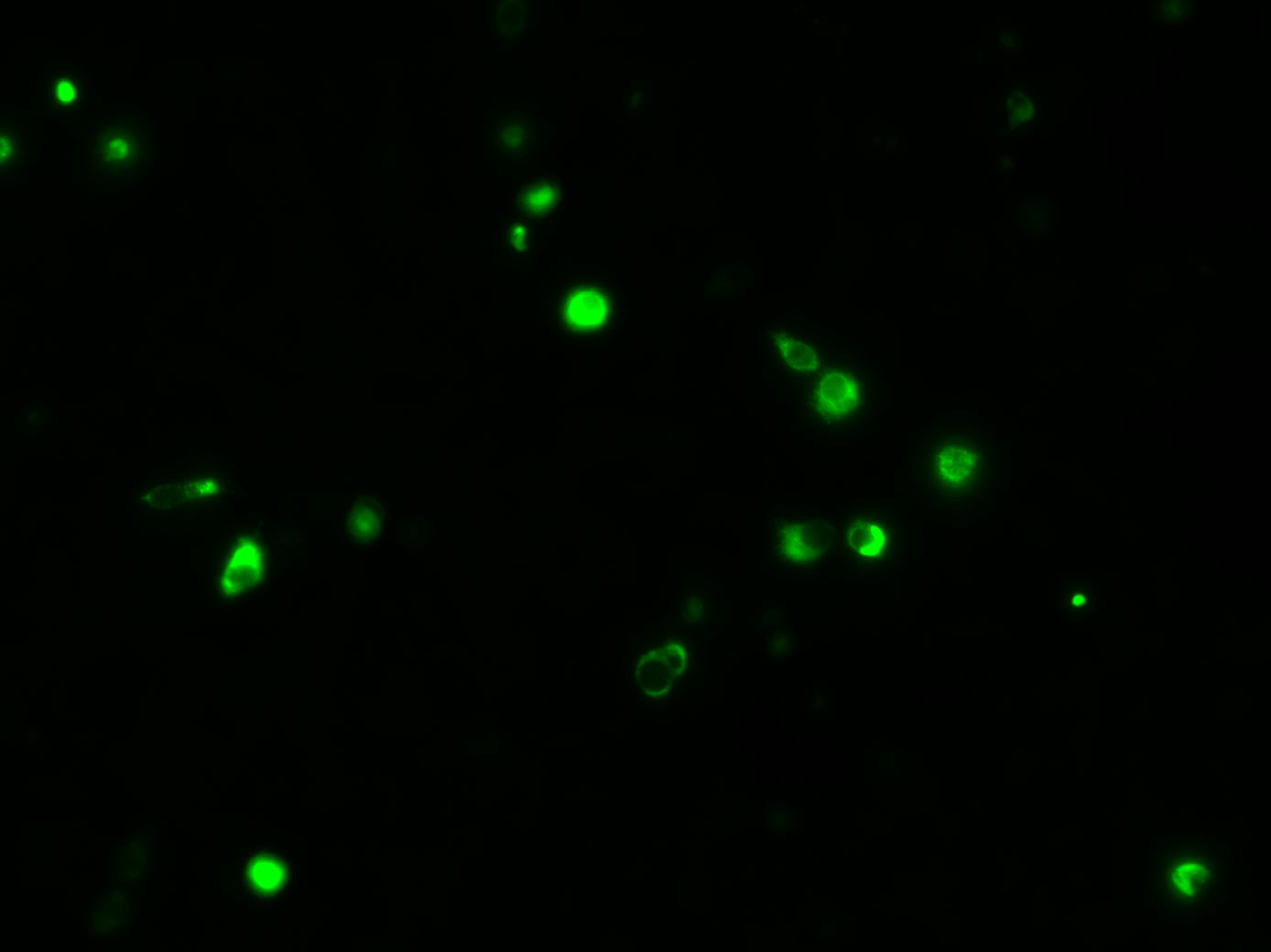

Supplement: S1 File — (ZIP) [file pntd.0012031.s001.zip › DB22-4_E_FITC.TIF]

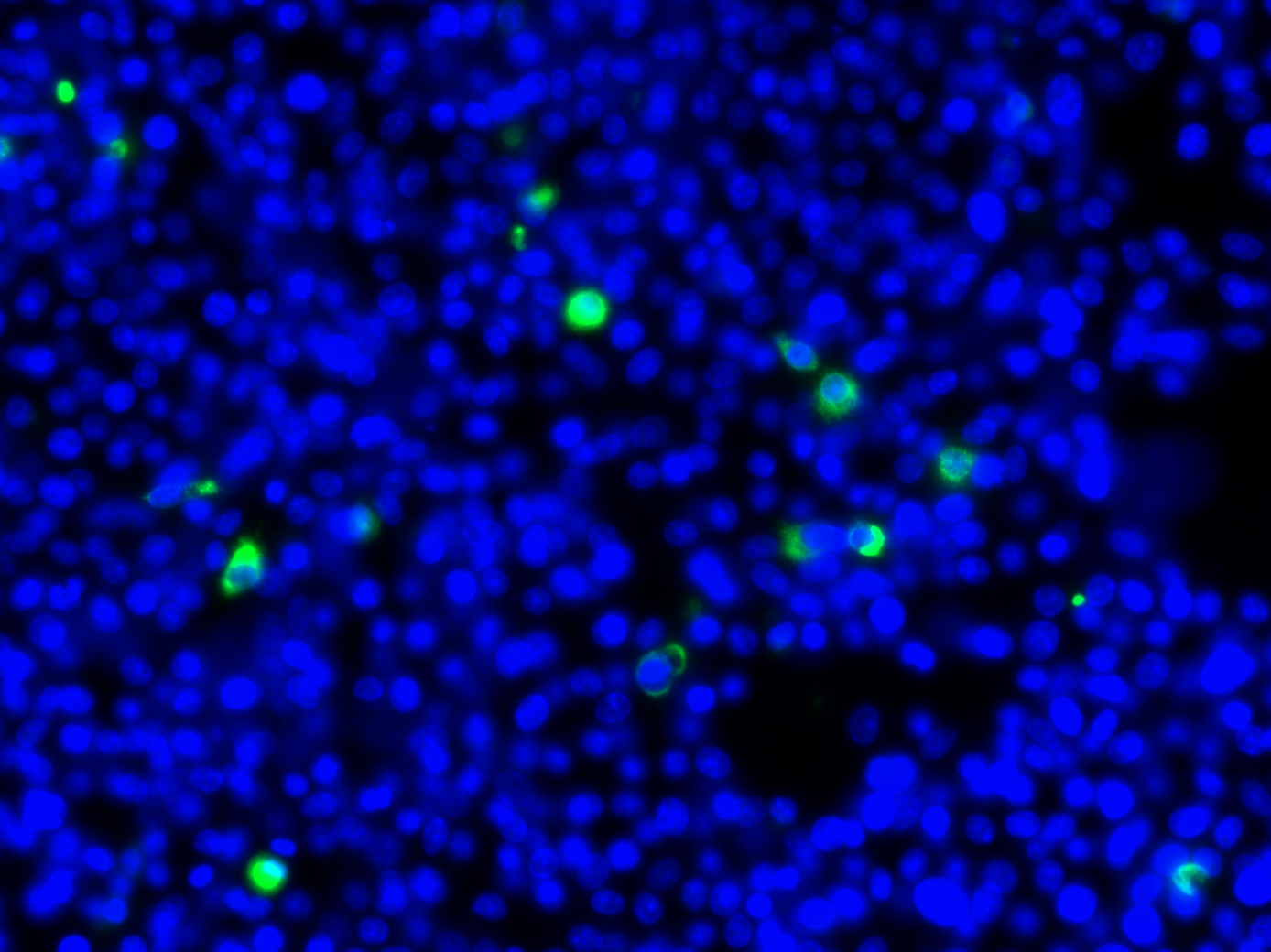

Supplement: S1 File — (ZIP) [file pntd.0012031.s001.zip › DB22-4_E_Merge.tif]

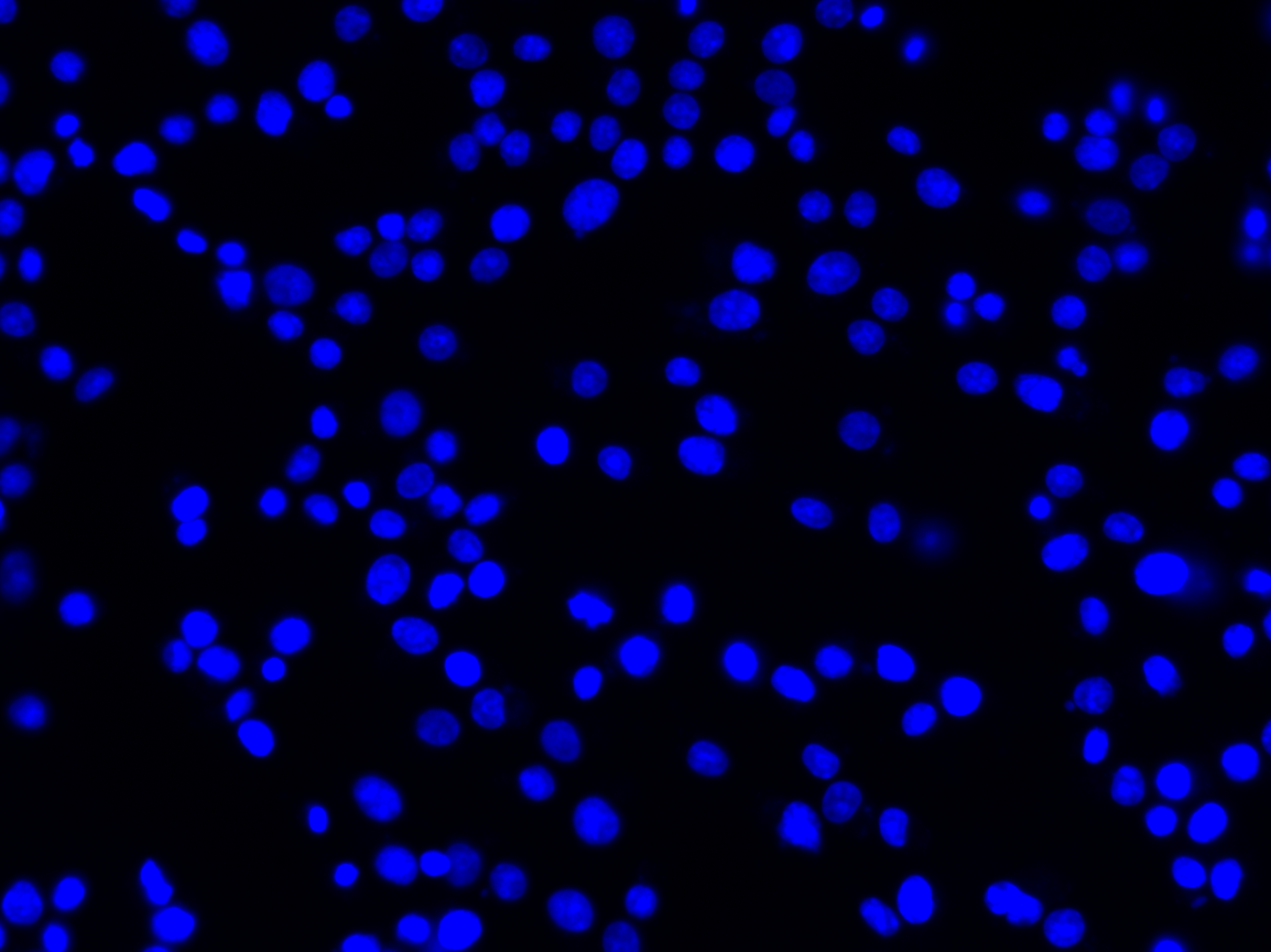

Supplement: S1 File — (ZIP) [file pntd.0012031.s001.zip › DB36-2_E_DAPI.TIF]

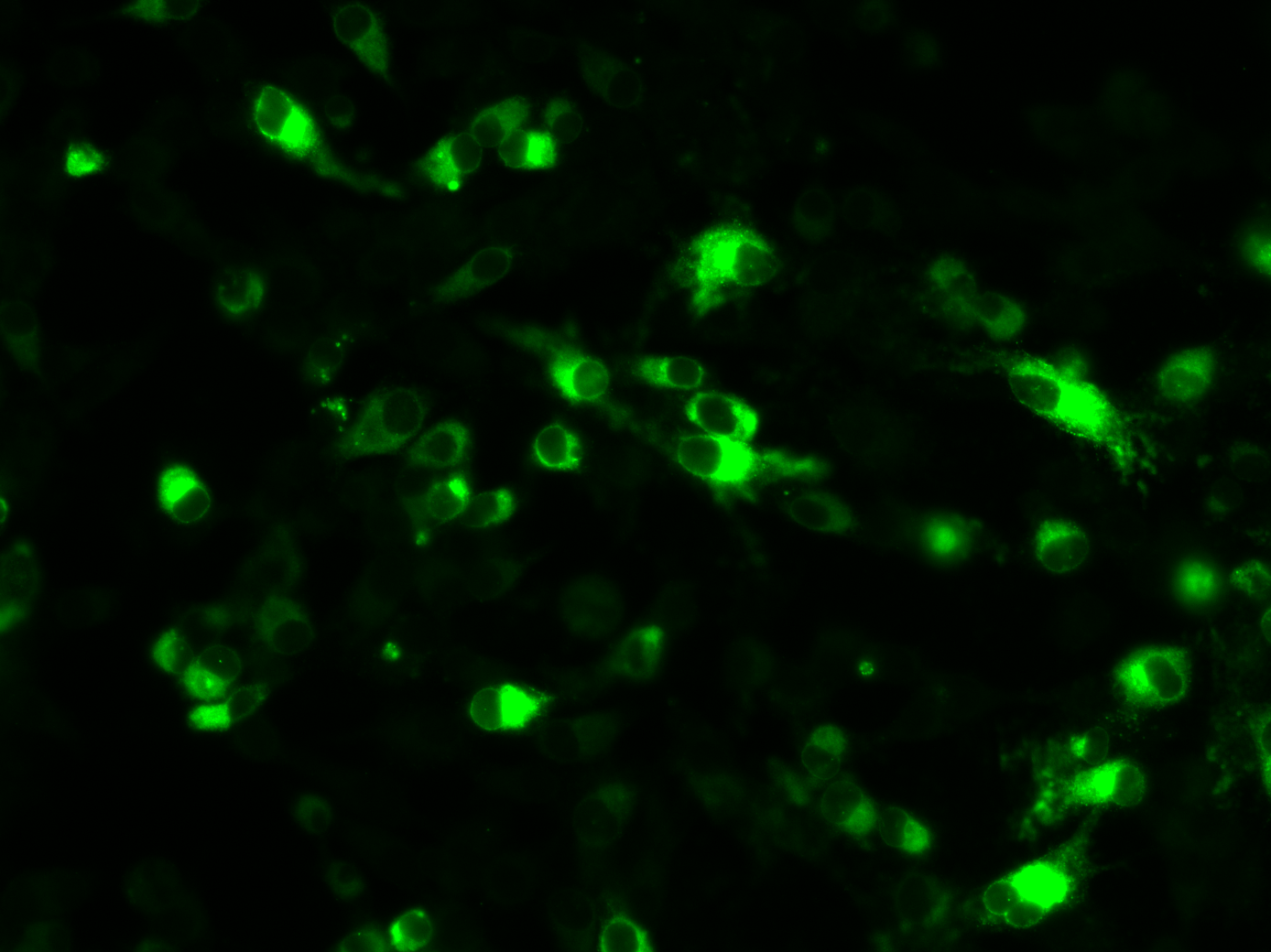

Supplement: S1 File — (ZIP) [file pntd.0012031.s001.zip › DB36-2_E_FITC.TIF]

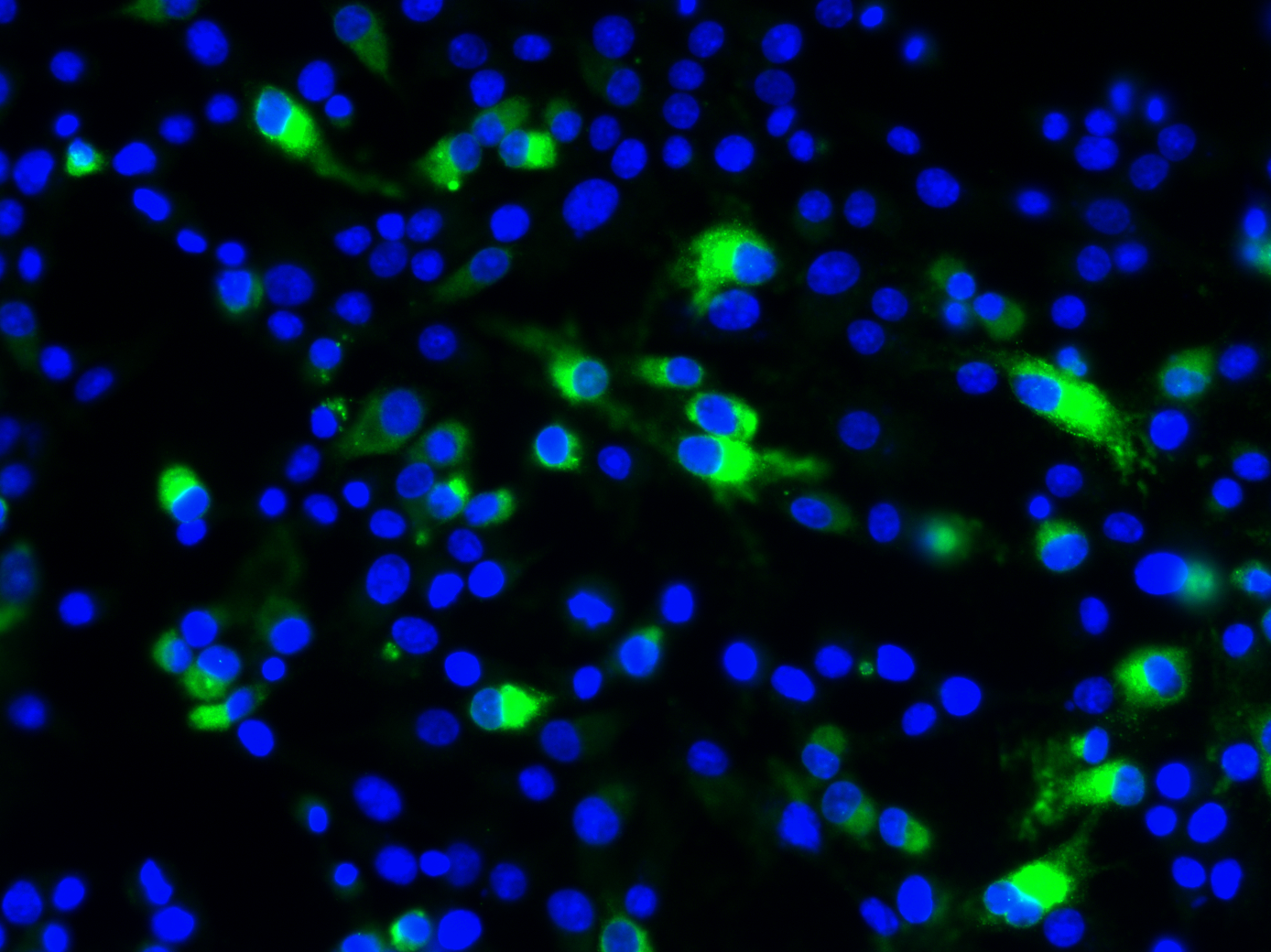

Supplement: S1 File — (ZIP) [file pntd.0012031.s001.zip › DB36-2_E_Merge.tif]

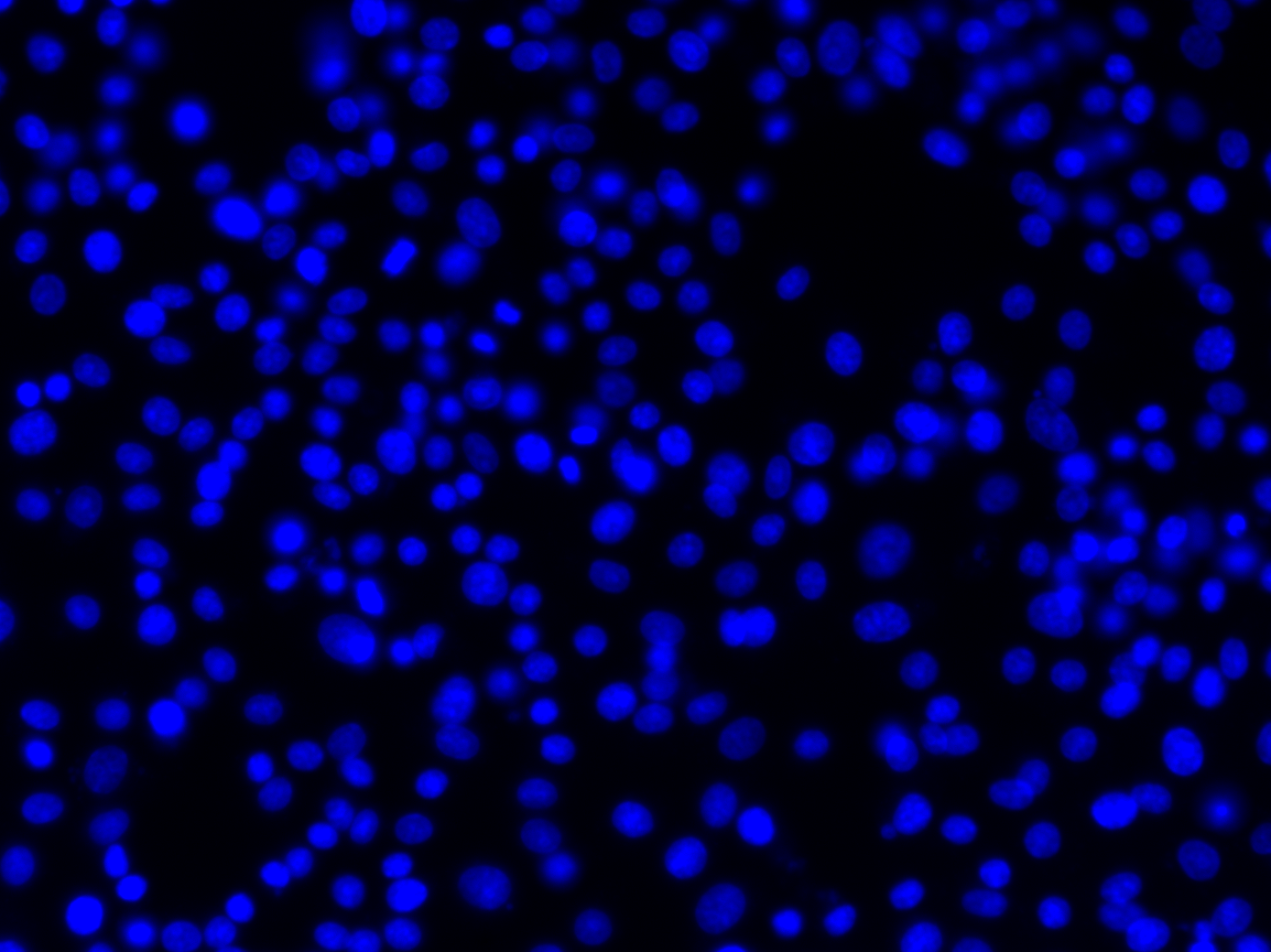

Supplement: S1 File — (ZIP) [file pntd.0012031.s001.zip › DB21-6_E_DAPI.TIF]

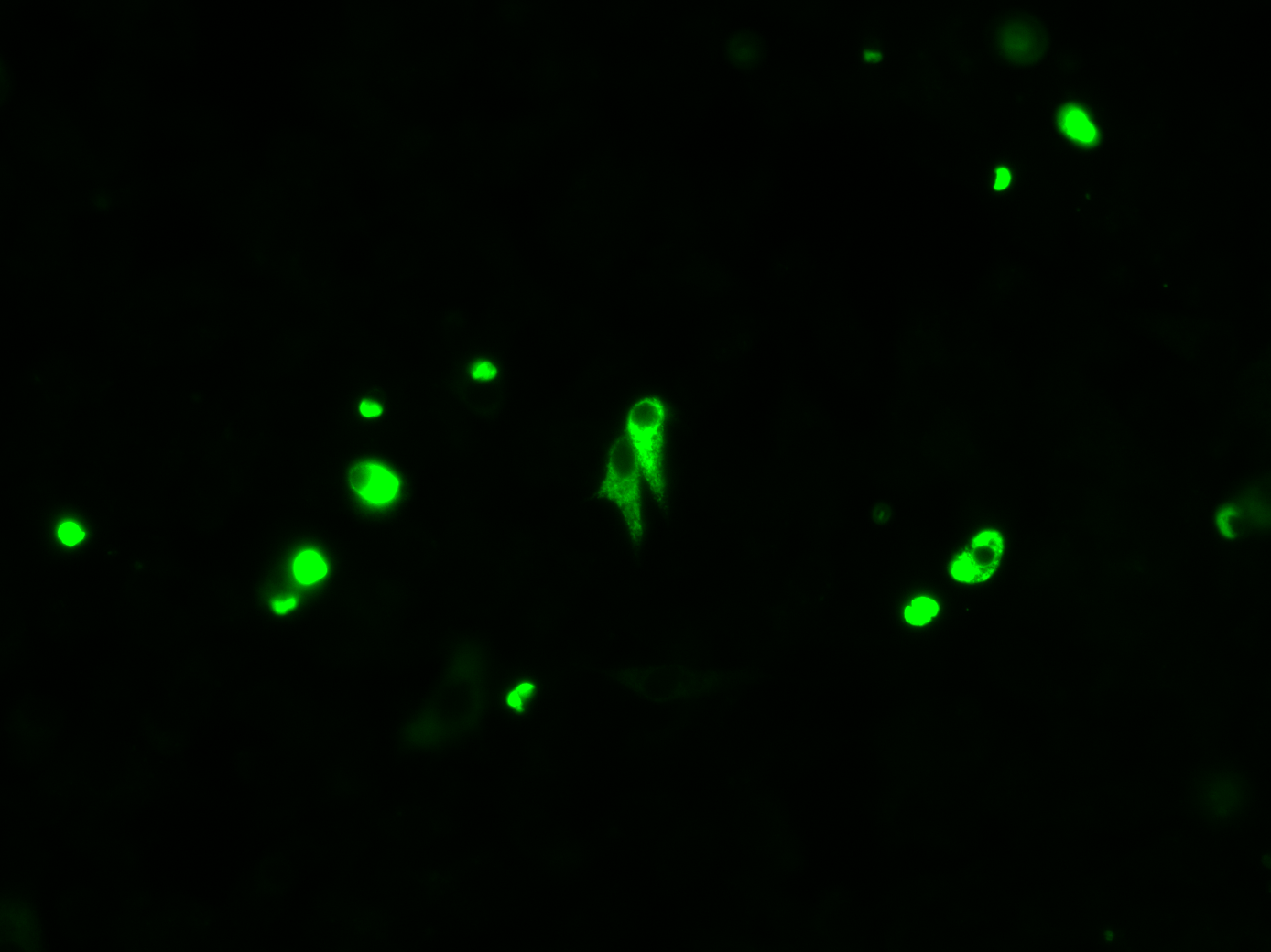

Supplement: S1 File — (ZIP) [file pntd.0012031.s001.zip › DB21-6_E_FITC.TIF]

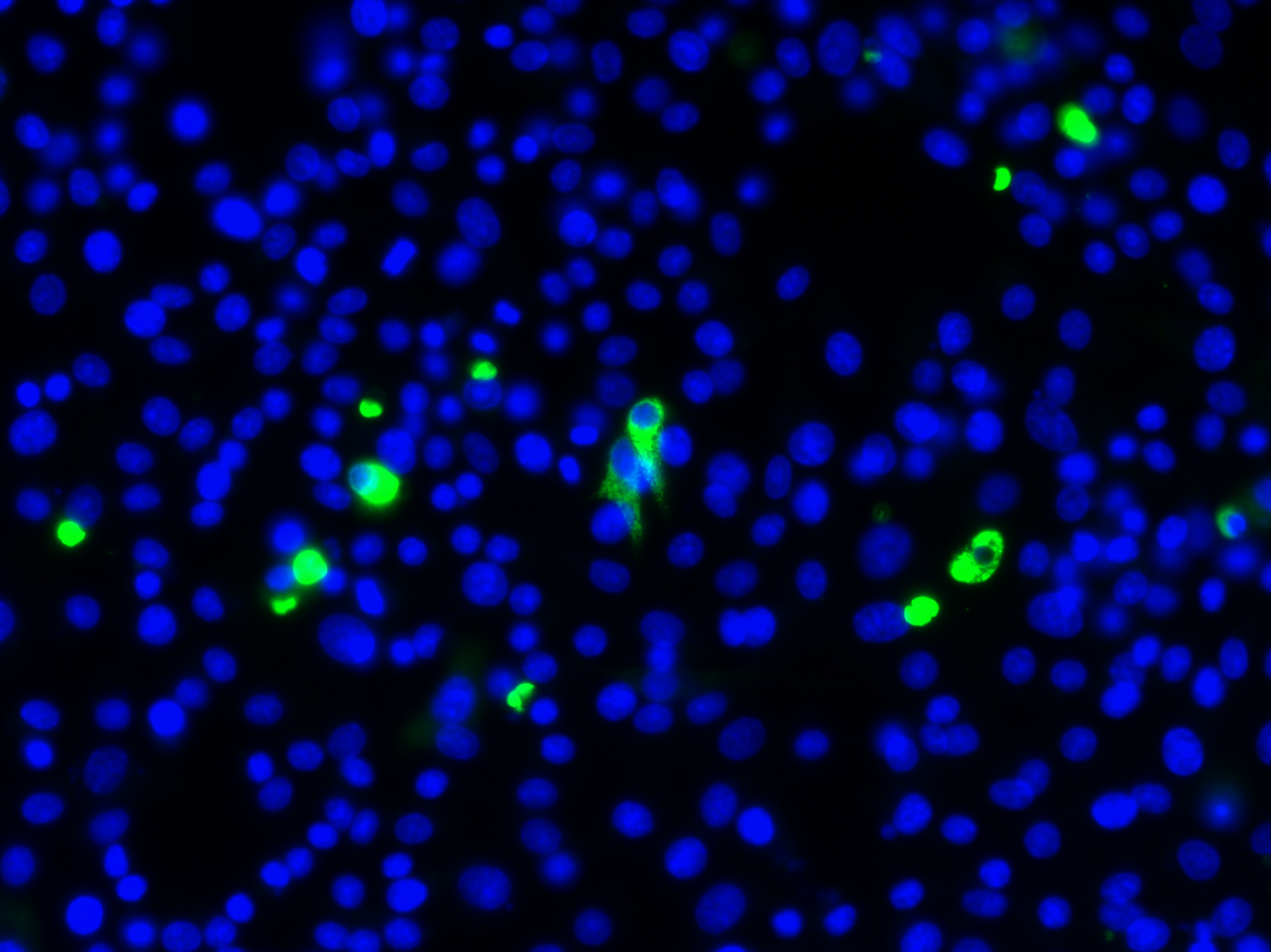

Supplement: S1 File — (ZIP) [file pntd.0012031.s001.zip › DB21-6_E_Merge.tif]
