# Supplementary material for: Correction: Development of a Humanized Antibody with High Therapeutic Potential against Dengue Virus Type 2
Source: PLoS Negl Trop Dis. 2024 Mar 13;18(3):e0012031. doi: 10.1371/journal.pntd.0012031 (PMC10936836; doi:10.1371/journal.pntd.0012031)
Supplement: S2 File — (PPTX) [file pntd.0012031.s002.pptx]

## Slide 1
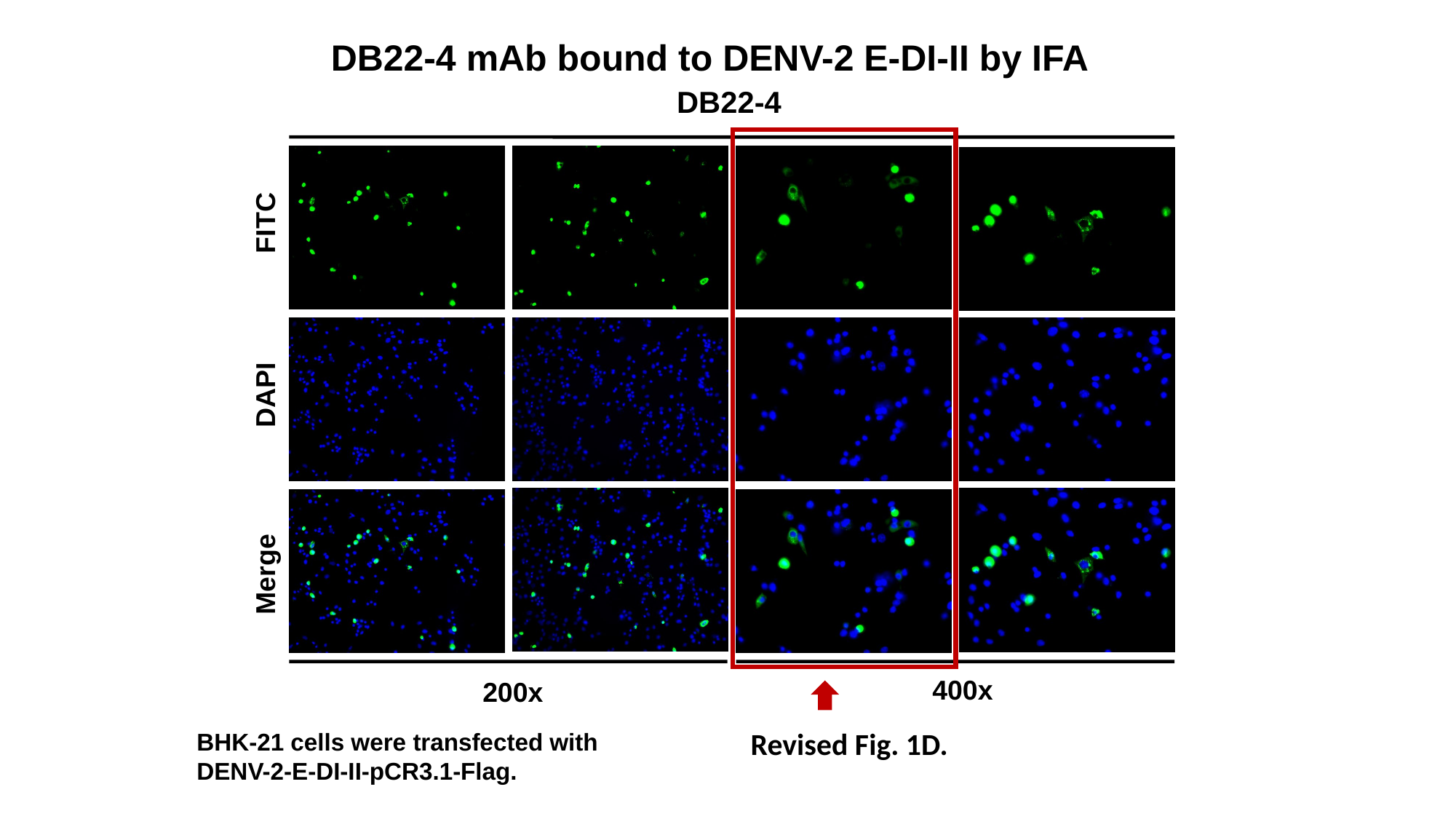

DB22-4 mAb bound to DENV-2 E-DI-II by IFA
DB22-4
FITC
DAPI
Merge
400x
200x
Revised Fig. 1D.
BHK-21 cells were transfected with DENV-2-E-DI-II-pCR3.1-Flag.

## Slide 2
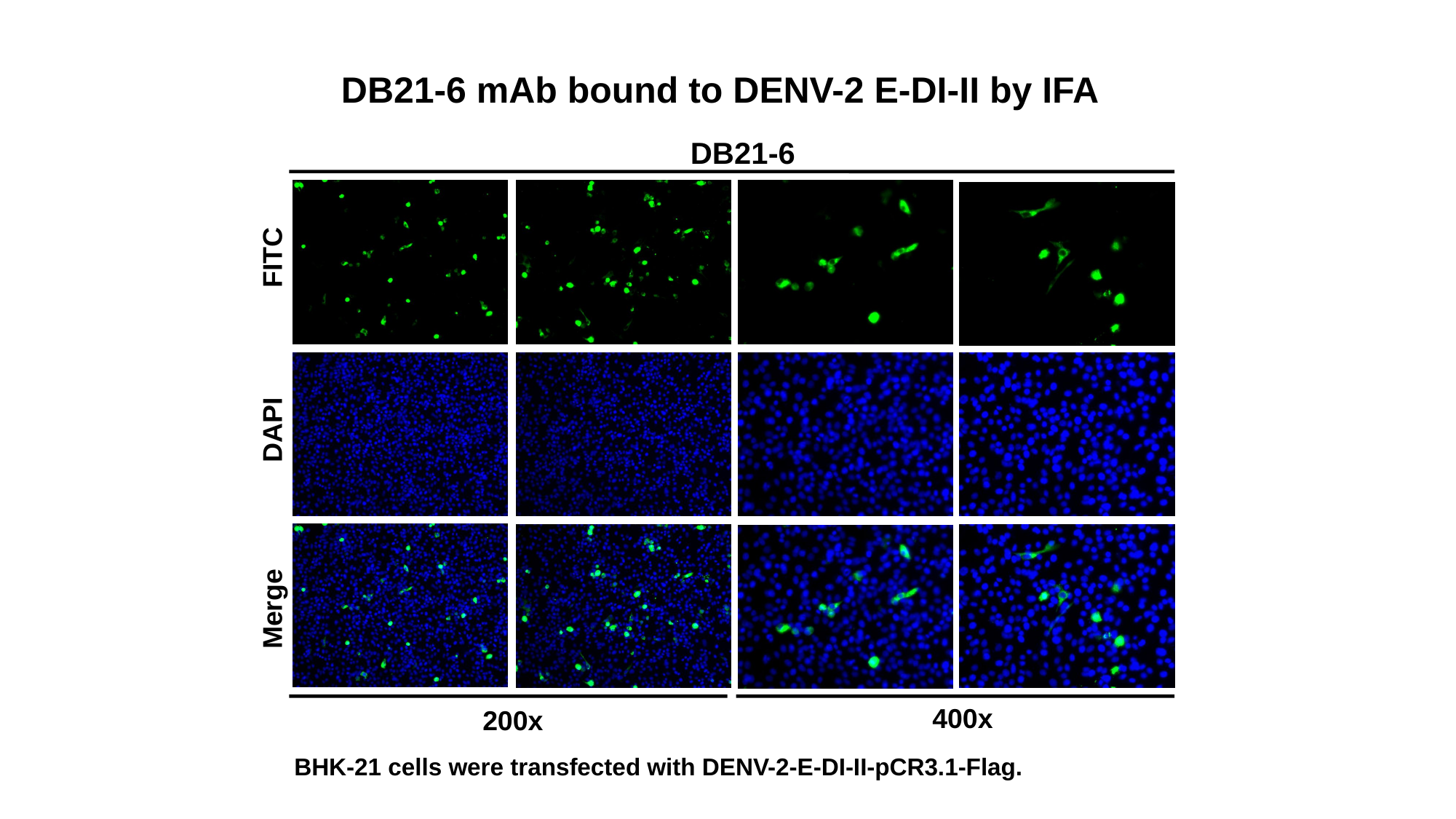

DB21-6 mAb bound to DENV-2 E-DI-II by IFA
DB21-6
FITC
DAPI
Merge
400x
200x
BHK-21 cells were transfected with DENV-2-E-DI-II-pCR3.1-Flag.
